# Inventory of the Seaweeds and Seagrasses of the Hawaiian Islands

**DOI:** 10.3390/biology12020215

**Published:** 2023-01-29

**Authors:** Alison R. Sherwood, Michael D. Guiry

**Affiliations:** 1 School of Life Sciences, University of Hawai‘i at Mānoa, Honolulu, HI 96822, USA; 2 AlgaeBase, Ryan Institute, University of Galway, H91 TK33 Galway, Ireland

**Keywords:** biodiversity, Chlorophyta, endemic, invasive species, Phaeophyceae, Rhodophyta

## Abstract

**Simple Summary:**

This inventory represents the first complete inventory of seaweeds (benthic brown, red, and green algae) and seagrasses from the Hawaiian Islands. We present taxonomic records compiled from the literature which include many recent descriptions of new species. Taxonomic records are accompanied by additional information allowing an assessment of the degree of molecular confirmation: whether the record has been verified by a match to DNA sequences from a type specimen, topotype sequences, or regional DNA sequences. In addition, taxa that have been identified solely based on morphology are indicated. In the face of numerous threats to biodiversity in the coming decades, it is hoped that this inventory will provide baseline data sets against which future changes may be compared.

**Abstract:**

This updated list is composed of a total of 661 records, which includes 71 brown algae, 450 red algae, 137 green algae, and three seagrasses, with an overall rate of endemism of 13.2%. Almost half (46.7%) of the Hawaiian records presented here are represented by at least one DNA sequence, while 16.3% are confirmed through a DNA sequence match to a topotype, and 6.7% are confirmed through a DNA sequence match to a type specimen. The data are presented in the context of the natural history of the Hawaiian Islands, which is heavily influenced by the volcanic hotspot origin of the archipelago in the middle of the Pacific Ocean, as well as the important cultural role of seaweeds and other marine plants in Hawai‘i, and the current threats to marine ecosystems, which include the introduction and proliferation of a number of invasive marine macroalgae.

## 1. Introduction

The Hawaiian Archipelago consists of eight major islands, together with numerous atolls and islets, positioned in the central North Pacific Ocean [1]. The islands were formed sequentially through hotspot volcanic activity on the Pacific Plate, such that islands to the northwest end of the archipelago are the oldest (i.e., Hōlanikū, or Kure Atoll, at over 28 million years [Ma]), and those at the southeastern end of the chain are the youngest notably Hawai‘i Island, at 0–0.6 Ma, with volcanic activity still producing new land [2]. The Archipelago extends for approximately 2400 km, and is extremely isolated, with the nearest continent (North America) being approximately 3900 km away [1].

The youngest and most southeastern islands of the archipelago, known as the Main Hawaiian Islands, are home to Hawai‘i’s permanent human population. The largest marine wildlife reserve in the world was established in 2006 to encompass the northwestern islands/atolls and surrounding waters of Hawai‘i (1,510,000 km^2^—approximately the size of Germany), named the Papahānaumokuākea Marine National Monument (PMNM). The largely uninhabited PMNM has deep cultural significance to Native Hawaiians, and research on the marine macroalgae from PMNM has indicated substantial undescribed biodiversity—e.g., [3,4,5,6,7,8]. Shorelines in Hawai‘i are largely determined by the geological processes that shaped the islands. Thus, initial volcanic activity yielded basaltic lava islands, and this feature still dominates the shorelines of Hawai‘i Island and nearby east Maui [9]. The gradual shifting of the Pacific Plate in a northwesterly direction, away from the volcanic hotspot, correlates with a slow geological degradation of the islands, with limestone caps, reefs, and sandy areas forming over time [9]; these features are progressively more prominent on the older islands.

The Hawaiian Archipelago extends from approximately 19° N (Hawai‘i Island) to 28° N (Hōlanikū), with the oldest Main Hawaiian island, Kaua‘i, positioned at 22° N [1]. Thus, the populated islands of Hawai‘i lie within the zone of the tropics, while many parts of PMNM are clearly subtropical by latitudinal definition, and their climate is generally considered to be subtropical [2]. The Hawaiian Islands experience two climatological periods annually, corresponding to a hot/dry season (May–October) and a cooler/wet season (November–April). Annual temperature variation is modest, with the capital city, Honolulu, experiencing an average annual temperature of 24 °C, ranging from 22.2 °C (February) to 25.8 °C (August) [2]. The surrounding ocean provides a strong buffer and has a moderating influence on air temperature; thus, although the Hawaiian Islands share approximately the same latitude as Cuba and Calcutta, India, the climate in Hawai‘i is not nearly as humid and hot; annual ocean temperatures range from 22.8 to 26.1 °C [2].

The Hawaiian Islands are well known as a location of high biological diversity and considerable endemism. Biogeographically, the Indo-West Pacific has had the greatest influence on the marine biodiversity of Hawai‘i, which is generally reported to have lower levels of endemism than the terrestrial biota, due to substantial levels of adaptive radiation in the terrestrial realm [2]. A 2003 compilation of the biota of Hawai‘i reported 578 seaweed species, of which 80 were endemic (13.8%), and 1197 species of marine fishes (153 endemic, or 12.8%), compared with 247 species of mosses (24 endemic, or 9.7%), 145 species of ferns (106 endemic, or 73.1%), 2142 species of angiosperms (896 endemic, or 41.8%), and 183 species of birds (63 endemic, or 34.4%), and with rates of endemism for most insect groups being much higher [10]. Sherwood [11] reported 2.3% endemism for the Hawaiian non-marine algal flora, as compiled from bibliographic records at that time.

An overview of the history of Hawaiian phycology was provided by Abbott [12], who described the key contributions to that point. The first photographic identification guide was published in 1979 [13]. Two books comprise the most up-to-date detailed flora of the Hawaiian Islands: Isabella Aiona Abbott’s 1999 *Marine Red Algae of the Hawaiian Islands* [12], and Abbott and John Huisman’s 2004 *Marine Green and Brown Algae of the Hawaiian Islands* [14], which are both based on morphological taxonomy. The 2007 publication by Huisman et al. of *Hawaiian Reef Plants* [9] united records of seaweeds and seagrasses and provided an updated photographic identification guide for the Islands, with information including the cultural relevance of algae, or *limu*. Studies in the early 2000’s began to examine Hawaiian seaweed systematics considering molecular data and phylogenetic reconstruction—a tool that has revolutionized understanding of algal evolutionary relationships worldwide. The higher-level taxonomy of many algal lineages has been clarified (e.g., order Peyssonneliales [15], order Sporolithales [16]), or, in some cases, newly discovered since that time (e.g., order Pihiellales [17], order Rhodachlyales [18]). Myriad changes at the lower taxonomic levels have also occurred, and these are, to the best of our abilities, reflected in this updated listing of Hawaiian marine algae and seagrasses.

Invasive seaweeds are now also common on some of Hawai‘i’s shorelines. Since the mid-20th century, macroalgae from elsewhere have arrived, either through hull-fouling, escape from aquaculture, or accidental introduction, and have made profound changes to some of the nearshore reefs of the Main Hawaiian Islands [9]. *Acanthophora spicifera* (M.Vahl) Børgesen is believed to have arrived via hull-fouling from a ship originating in Guam, while species such as *Gracilaria salicornia* (C.Agardh) E.Y.Dawson, *Hypnea musciformis* (Wulfen) J.V.Lamouroux, *Eucheuma denticulatum* (N.L.Burman) Collins and Hervey, and *Kappaphycus alvarezii* (Doty) L.M.Liao were probable escapees from aquaculture or industrial attempts [9]. Native or cryptogenic species are also known to be problematic in Hawai‘i—examples include the green seaweed *Dictyosphaeria cavernosa* (Forsskål) Børgesen in Kāne‘ohe Bay on O‘ahu, the green seaweed *Avrainvillea lacerata* J.Agardh [generally referred to as *A. amadelpha* (Montagne) A.Gepp and E.S.Gepp in the local literature], and the newly discovered red seaweed *Chondria tumulosa* A.R.Sherwood and Huisman, which is wreaking havoc at several atolls in PMNM [9,19]. A second species of *Avrainvillea*, *A*. cf. *erecta* A.Gepp and E.S.Gepp was also recently reported from urbanized estuaries of the Main Hawaiian Islands [20]. Impacts of these newcomers are variable, with some demonstrating clear and immediate negative impacts on the native flora and fauna (e.g., *C. tumulosa*), while others will need to be evaluated for a longer period in order to comprehend these effects (e.g., *A. erecta*).

Marine algae play an important cultural role to Native Hawaiians. Due to religious restrictions placed on foods that women could eat (ca. A.D. 1200), women became specialists of shoreline algae and invertebrates, which were available to them, and the Hawaiian term *limu* has come to mean “edible algae”, although the original term was somewhat broader and included some simply constructed corals, liverworts, mosses, lichens, and fungi [12]. Hawaiian names were applied to plants that could be used as medicine or food, or in other ways [21], and some of the best known of these practices are given by Huisman et al. [9]. Seaweed and algae research generally receives a very positive reaction from the present day population of Hawai‘i—as an example, Hawai‘i’s Governor (David Ige) proclaimed 2022 as “The Year of the Limu”, which is accompanied by numerous public events celebrating and educating about Hawaiian marine algae (https://governor.hawaii.gov/newsroom/dlnr-news-release-year-of-the-limu-recognizes-importance-of-indigenous-species-of-plants-and-algae/, accessed on 2 October 2022).

The objective of the current study is to compile and summarize records of seaweeds (and seagrasses, of which there are only a few) from the Hawaiian Islands, which will allow comparisons to other floras, and provide a point-in-time snapshot of current understanding of the flora. Despite many decades of research on the Hawaiian algal flora by a number of notable phycologists, and published inventories for more narrowly defined parts of the flora (e.g., [12] for Hawaiian marine red algae; [11] for the Hawaiian non-marine algae; [22] for marine algae of PMNM), no current inventory exists for Hawaiian marine macroalgae and seagrasses. We seek to rectify that through the present study.

## 2. Materials and Methods

**Figure 1:**
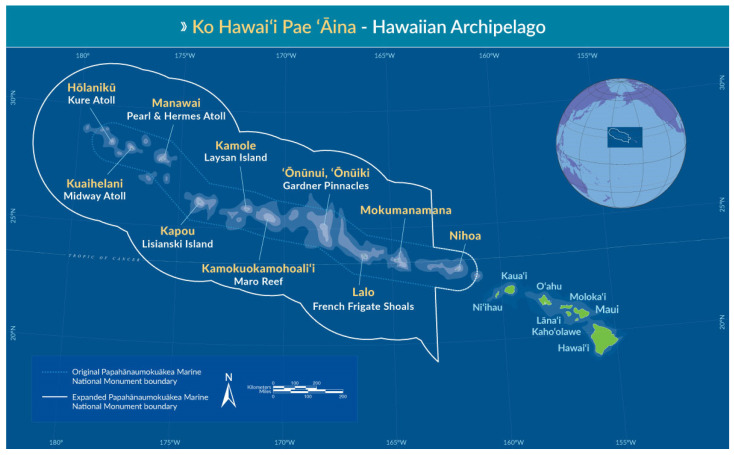
Map of the Hawaiian Archipelago (courtesy of NOAA-PMNM). The map includes the Main Hawaiian Islands (shown in green) as well as the Papahānaumokuākea Marine National Monument (with the extent of the Monument indicated with a white outline). Marine algal records from both regions are included in the inventory.

An initial set of records was compiled from AlgaeBase (on 18 March 2022) as algal and seagrass taxa that included the Hawaiian Islands in their distribution. This initial list was then hand-curated to remove Cyanobacteria and non-marine records. Other records were added from the literature not included in AlgaeBase, such as from recent theses and dissertations, and from in-press manuscripts known to the authors. In particular, publications reporting DNA sequence data are included to provide further information on the level of confidence in molecular confirmation. A second AlgaeBase download was performed on 10 December 2022 to check for records added since the initial download. Entries include records from both the Main Hawaiian Islands (Ni‘ihau, Kaua‘i, O‘ahu, Moloka‘i, Maui, Lāna‘i, Kaho‘olawe, and Hawai‘i) and the Papahānaumokuākea Marine National Monument (Hōlanikū [Kure Atoll], Kuaihelani [Midway Atoll], Manawai [Pearl and Hermes Atoll], Kapou [Lisianski Island], Kamole [Laysan Island], Kamokuokamohoali‘i [Maro Reef], ‘Ōnūnui and ‘Ōnūiki [Gardner Pinnacles], Lalo [French Frigate Shoals], Mokumanamana, and Nihoa), as well as minor islands and islets (Figure 1).

The list includes taxonomic information for each record including major lineage (e.g., phylum), order, family, genus, and species (and, where appropriate, subspecific taxon) (Table 1). The table column “regionally reported as” is used to indicate instances where a different name is or has been used to refer to the taxon in the Hawaiian Islands and includes taxonomic synonyms as well as common and widespread misidentifications. Whether a taxon is endemic to the Hawaiian Islands is indicated (as yes, no, or maybe). Four subsequent columns give information about the degree of molecular confirmation (where present), and whether the taxon has been verified based on morphological comparisons: “Morphology” (identification is based on morphological examination), “Regional DNA Sequence” (identification based on one or more DNA sequences generated from Hawaiian material and compared to sequences from elsewhere), “Topotype Sequence Match” (sequences of Hawaiian material have been shown to reasonably match those from topotype material), or “Type Sequence Match” (sequences of Hawaiian material have been shown to reasonably match that of a type specimen for that taxon). Hawaiian sequences were considered to represent the topotype locality if they were from the same island. Finally, one or more representative references for each species record are listed (not a comprehensive list of all records), and a column for taxonomic notes is included. Taxa listed as doubtful records are still included in the totals presented, until such time as they are discounted as members of the Hawaiian flora.

As for any inventory, the current listing represents a point-in-time and will change as more new species and genera are described (we are aware of a number of these in progress at the time of writing but are not yet accepted for publication), and other kinds of taxonomic proposals (e.g., synonymies or removal of records) are put forward.

## 3. Results

**Figure 2:**
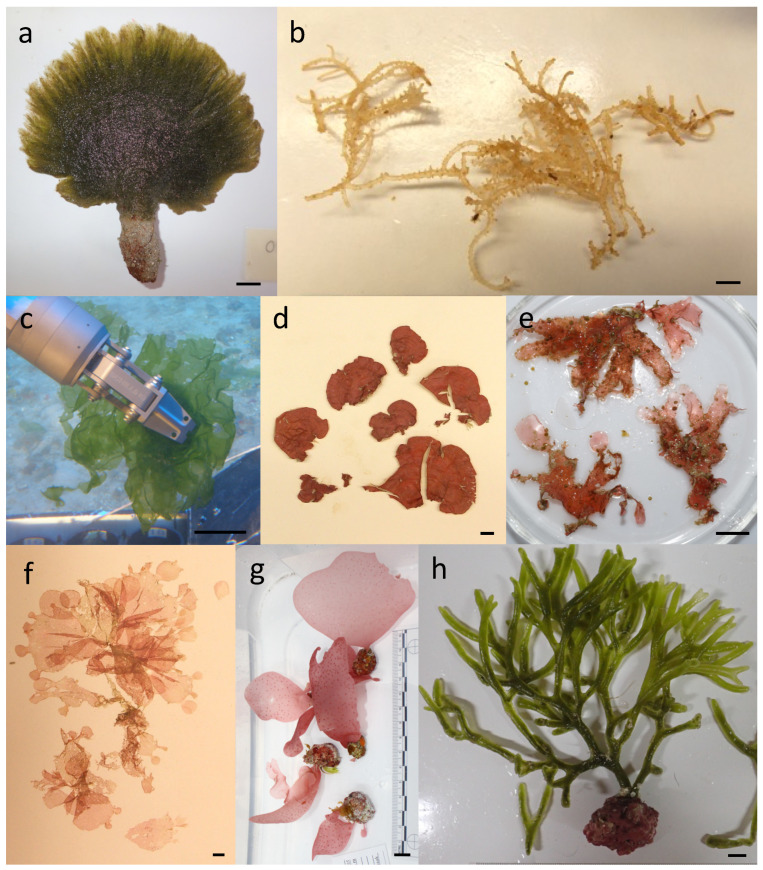
Select species of Hawaiian marine macroalgae, including some recently discovered and/or described taxa. (**a**). *Avrainvillea erecta*, a green alga recently reported as a new introduction to Hawaiian waters. (**b**). *Chondria tumulosa*, a recently described red alga that forms nuisance blooms in PMNM. (**c**). *Ulva ohiohilulu*, a mesophotic green alga described from Hawai‘i (**d**). *Sonderophycus copusii*, a peyssonnelioid red alga known only from mesophotic depths in PMNM. (**e**). *Haraldiophyllum hawaiiense*, a red alga described in 2020 from 81 to 93 m depth in PMNM (**f**). *Martensia lauhiekoeloa*, a red alga described in 2019 from 61 to 67 m depth in PMNM (**g**). *Psaromenia laulamaula*, a red alga from 83 to 94 m in PMNM (**h**). *Codium fragile*, a widespread green alga known also from Hawai‘i. Scale bar = 1 cm (all but (**c**)); 5 cm (**c**).

A total of 661 marine algal and seagrass taxa are included in this Hawaiian inventory (Table 1, Figure 2). These records include 71 Phaeophyceae, or brown algae (representing 11 orders, 16 families, and 33 genera), 450 Rhodophyta, or red algae (23 orders, 58 families, and 197 genera), 137 Chlorophyta/Prasinodermatophyta, or green algae (7 orders, 23 families, and 41 genera), and 3 Tracheophyta, or seagrasses (1 order, 2 families, and 2 genera). The Hawaiian marine flora as currently inventoried is 13.2% endemic, with values of 33.3% (seagrasses), 2.8% (brown algae), 6.6% (green algae), and 16.7% (red algae). 

The vast majority (99.5%) of these records are based, at least in part, on morphological identifications. Approximately 46.7% of the flora is represented by at least one DNA sequence generated from Hawaiian material, meaning that 53.3% of the flora remains to be characterized using molecular tools, at any level. Seagrasses have the highest percentage of the flora represented by at least one DNA sequence (66.7%, albeit only three taxa comprise this component of the flora), followed by red algae (53.8%), brown algae (32.4%), and green algae (30.7%). A total of 16.3% of the records in the inventory has been shown to have a reasonably close match to a topotype, with values ranging from 0% (seagrasses), to 8.8% (green algae), 16.9% (brown algae), and 18.7% (red algae). A relatively small percentage of records (6.7%) has been confirmed through a reasonably close match to a sequence derived from type material (which includes recently described species for which DNA sequence data have been provided as part of the description); these values range from 0% (seagrasses), to 4.4% (green algae), 5.6% (brown algae), and 7.6% (red algae).

## 4. Discussion

We present the first comprehensive inventory of Hawaiian marine algae and seagrasses combining both morphological and molecular data and provide data on the degree of endemism of the flora, as well as degree of certainty in identification for each record. Despite having several very thorough morphological compendia available for various lineages of the Hawaiian flora, no single compilation of species existed. The most recent compendium of the red, green, and brown marine algae of Hawaii reported a total of 512 species (343 red algae, 107 green algae, and 62 brown algae [12,14]), which compares with the 661 subgeneric taxa (450 red algae, 137 green algae, and 71 brown algae, plus 3 seagrasses) in our current inventory. With 47% of records represented by at least one DNA sequence generated from Hawaiian material, a large amount of survey work and inventory curation awaits additional sequencing efforts. However, much recent taxonomic and systematic revision has occurred for the flora, which further emphasizes the need for the present inventory. Future updates are certain as this work continues, particularly with respect to new species descriptions from the mesophotic flora. 

Degree of confidence in identification is becoming increasingly recognized as an important part of reporting in taxonomic inventories, lists and checklists—e.g., [23]. Variation in confidence of identification is an unavoidable side-effect of combining records from historical sources, which largely relied on a strictly morphological identification approach, with more recent works that include DNA sequence comparisons. Moreover, interpretation of DNA sequence comparisons is being undertaken with a higher level of scrutiny than when the tool first become available to phycologists, when sequences deposited in public databases may or may not have represented the labeled taxon due to sequence quality issues or misidentification (which could be due to several factors, further described below). For example, highest confidence can be placed on an identification when one or more DNA sequences matches or nearly matches a sequence derived from type material of that taxon. These instances are rare given that most taxa are not represented by DNA sequences generated from their type specimens, but 6.7% of the records in the current inventory have indeed been verified at that level, primarily as a result of new species descriptions from the Hawaiian flora that include molecular characterization. The next highest level of confidence in identification stems from an often-used proxy for type sequences—topotype sequence data, or sequences derived from specimens collected from (or relatively close to) the type locality. Topotype sequences, however, may in fact be representative of other taxa that cohabit that locality, or other taxa found in nearby areas, if the topotype locality is interpreted too broadly. The third highest level of confidence in identification stems from sequences generated from regionally collected material that was morphologically identified as belonging to a specific taxon or had one or more molecular markers reasonably matching a labeled sequence from a public database of that taxon. Strictly morphological identifications may be reasonably accurate for some groups of marine algae and seagrasses but are known to be rife with potential for misidentification due to several factors.

Marine algal taxonomy traditionally relied upon comparison of morphological characters, which remains a critical component of marine macroalgal identification, but can have several disadvantages [24]. For example, features associated with sexual reproduction are often necessary to discern species. Yet, these can be missing from specimens. 

**Table 1:** Inventory of Hawaiian marine macroalgae and seagrasses.

Major Lineage (e.g., Phylum)	Order	Family	Genus	Species	Regionally Reported As ^1^	Endemic ^2^	Morphology ^2^	Regional DNA Sequence ^2^	Topotype Sequence Match ^2^	Type Sequence Match ^2^	References ^3^	Notes
Phaeophyceae	Asterocladales	Asterocladaceae	*Asterocladon* D.G.Müller, E.R.Parodi and A.F.Peters	*A. rhodochortonoides* (Børgesen) S.Uwai, C.Nagasato, T.Motomura and K.Kogame	*Asteronema rhodochortonoides* (Børgesen) D.G.Müller and Parodi	N	Y	N	N	N	[14]	
	Desmarestiales	Desmarestiaceae	*Desmarestia* J.V.Lamouroux	*D. ligulata* (Stackhouse) J.V.Lamouroux		N	Y	N	N	N	[14,22]	
	Dictyotales	Dictyotaceae	*Dictyopteris* J.V.Lamouroux	*D. australis* (Sonder) Askenasy		N	Y	N	N	N	[9,14,22]	
				*D. plagiogramma* (Montagne) Vickers		N	Y	N	N	N	[9,14,22]	
				*D. repens* (Okamura) Børgesen		N	Y	N	N	N	[14,22]	
			*Dictyota* J.V.Lamouroux	*D. acutiloba* J.Agardh		N	Y	Y	Y	N	[9,14,22,23]	
				*D. bartayresiana* J.V.Lamouroux		N	Y	N	N	N	[14,22]	
				*D. ceylanica* Kützing		N	Y	N	N	N	[9,14,22]	
				*D. ciliolata* Sonder ex Kützing		N	Y	N	N	N	[14,22]	
				*D. flabellata* (Collins) Setchell and N.L.Gardner		N	Y	N	N	N	[14]	
				*D. friabilis* Setchell		N	Y	Y	Y	N	[9,14,22,23]	
				*D. implexa* (Desfontaines) J.V.Lamouroux	*D. divaricata* J.V.Lamouroux	N	Y	N	N	N	[13]	
				*D. sandvicensis* Sonder		N	Y	Y	Y	N	[9,14,22,23]	
				*D. stolonifera* E.Y. Dawson		N	Y	N	N	N	[14,22]	
			*Distromium* Levring	*D. flabellatum* Womersley		N	Y	N	N	N	[9,14,22]	
			*Lobophora* J.Agardh	*L. obscura* (Dickie) C.W.Vieira, De Clerck and Payri	*L. crassa* Z.Sun, P.E.Lim and H.Kawai, *L. variegata* (J.V.Lamouroux) Womersley ex E.C.Oliveira	N	Y	Y	Y	Y	[9,14,22,25]	Hawaiian specimens are not *L. variegata*, and are presumably now known as *L. crassa* [25]
			*Newhousia* Kraft, G.W.Saunders, I.A.Abbott and Haroun	*N. imbricata* Kraft, G.W.Saunders, I.A.Abbott and Haroun		N	Y	Y	Y	Y	[9,23,26,27]	
			*Padina* Adanson	*P. australis* Hauck		N	Y	Y	N	N	[9,14,22,28]	
				*P. boergesenii* Allender and Kraft		N	N	Y	N	N	[22]	
				*P. boryana* Thivy		N	Y	N	N	N	[14,22]	
				*P. gymnospora* (Kützing) Sonder		N	Y	N	N	N	[14,22]	
				*P. japonica* Yamada		N	Y	N	N	N	[13,29]	
				*P. maroensis* Ni-Ni-Win, I.A.Abbott and H.Kawai		Y	Y	Y	Y	Y	[28]	
				*P. melemele* I.A.Abbott and Magruder		N	Y	Y	N	N	[9,14,22,23,28,30]	
				*P. moffittiana* Abbott and Huisman		N	Y	Y	Y	N	[14,22,30]	
				*P. sanctae-crucis* Børgesen		N	Y	Y	N	N	[9,14,22]	
				*P. thivyae* Doty and Newhouse		N	Y	N	N	N	[9,14]	
			*Spatoglossum* Kützing	*S. macrodontum* J.Agardh		N	Y	N	N	N	[9,14]	
				*S. solieri* (Chauvin ex Montagne) Kützing		N	Y	N	N	N	[13]	
			*Stypopodium* Kützing	*S. flabelliforme* Weber Bosse	*S. hawaiiensis* (Doty and Newhouse) I.A.Abbott, *Zonaria hawaiiensis* Doty and Newhouse	N	Y	Y	N	N	[9,14,22]	
	Discosporangiales	Discosporangiaceae	*Discosporangium* Falkenberg	*D. mesarthrocarpum* (Meneghini) Hauck		N	Y	N	N	N	[14]	
	Ectocarpales	Acinetosporaceae	*Feldmannia* Hamel	*F. indica* (Sonder) Womersley and A.Bailey	*Hincksia indica* (Sonder) J.Tanaka	N	Y	N	N	N	[9,14,22]	
				*F. irregularis* (Kützing) Hamel		N	Y	N	N	N	[14,22]	
				*F. lebelii* (Areschoug ex P.Crouan and H.Crouan) Hamel		N	Y	N	N	N	[9,14,22]	
				*F. mitchelliae* (Harvey) H.-S.Kim	*Hincksia mitchelliae* (Harvey) P.C.Silva	N	Y	N	N	N	[14,22]	
			*Hincksia* J.E.Gray	*H. conifera* (Børgesen) I.A.Abbott		N	Y	N	N	N	[22]	
			*Pylaiella* Bory	*P. littoralis* (Linnaeus) Kjellman		N	Y	N	N	N	[14]	
		Chordariaceae	*Kuetzingiella* Kornmann	*K. elachistiformis* (Heydrich) M.Balakrishnan and Kinkar		N	Y	N	N	N	[14,22]	
			*Nemacystus* Derbès and Solier	*N. decipiens* (Suringar) Kuckuck		N	Y	N	N	N	[9,14,22]	
		Scytosiphonaceae	*Chnoospora* J.Agardh	*C. minima* (Hering) Papenfuss		N	Y	Y	N	N	[9,14,22,31]	
			*Colpomenia* (Endlicher) Derbès and Solier	*C. sinuosa* (Mertens ex Roth) Derbès and Solier		N	Y	Y	Y	N	[9,14,22,32,33]	
				*C. tuberculata* D.A.Saunders		N	Y	N	N	N	[9,14,22]	
			*Hydroclathrus* Bory	*H. clathratus* (C.Agardh) M.Howe		N	Y	N	N	N	[9,14,22]	
				*H. tilesii* (Endlicher) Santiañez and M.J.Wynne	*H. stephanosorus* Kraft	N	Y	Y	N	N	[34]	
				*H. tenuis* C.K.Tseng and Lu Baroen		N	N	Y	Y	N	[34]	
				*H. tumulis* Kraft and Abbott		N	Y	N	N	N	[9,14,22]	
			*Petalonia* Derbès and Solier	*P. binghamiae* (J.Agardh) K.L.Vinogradova	*Endarachne binghamiae* J.Agardh	N	Y	N	N	N	[14]	Hawaiian specimens reported as *P. binghamiae* are most likely *P. tatewakii* [31]
				*P. tatewakii* Kogame and Kurihara		Y	Y	Y	Y	Y	[31]	
			*Pseudochnoospora* Santiañez, G.Y.Cho and Kogame	*P. implexa* (J.Agardh) Santiañez, G.Y.Cho and Kogame	*Chnoospora implexa* J.Agardh	N	Y	N	N	N	[9,14]	
			*Rosenvingea* Børgesen	*R. endiviifolia* (Martius) M.J.Wynne	*R. intricata* (J.Agardh) Børgesen	N	Y	N	N	N	[9,14]	
				*R. orientalis* (J.Agardh) Børgesen		N	Y	N	N	N	[9,14]	
	Fucales	Sargassaceae	*Sargassum* C.Agardh	*S. aquifolium* (Turner) C.Agardh	*S. echinocarpum* J.Agardh	N	Y	Y	N	N	[9,14,22,35]	
				*S. muticum* (Yendo) Fensholt		N	Y	N	N	N	[9,14]	
				*S. obtusifolium* J.Agardh	*S. hawaiiense* Doty and Newhouse	N	Y	Y	Y	N	[9,14,22,35]	
				*S. oligocystum* Montagne		N	Y	N	N	N	[36]	
				*S. piluliferum* (Turner) J.Agardh		N	Y	N	N	N	[22]	
				*S. polyphyllum* J.Agardh		N	Y	Y	Y	N	[9,14,22,35]	
			*Turbinaria* J.V.Lamouroux	*T. ornata* (Turner) J.Agardh		N	Y	Y	N	N	[9,14,22,37]	
	Ralfsiales	Mesosporaceae	*Mesospora* Weber Bosse	*M. pangoensis* (Setchell) Chihara and J.Tanaka	*Hapalospongidion pangoense* (Setchell) Hollenberg, *Ralfsia pangoensis* Setchell	N	Y	N	N	N	[14]	
				*M. schmidtii* Weber Bosse		N	Y	N	N	N	[38]	
		Neoralfsiaceae	*Neoralfsia* P.-E.Lim and H.Kawai	*N. expansa* (J.Agardh) P.-E.Lim and H.Kawai ex Cormaci and G.Furnari	*Ralfsia expansa* (J.Agardh) J.Agardh	N	Y	N	N	N	[9,14]	
	Scytothamnales	Asteronemataceae	*Asteronema* Delépine and Asensi	*A. breviarticulatum* (J.Agardh) Ouriques and Bouzon	*Giffordia breviarticulata* (J.Agardh) Doty and I.A.Abbott, *Hincksia breviarticulata* (J.Agardh) P.C.Silva	N	Y	Y	N	N	[9,14,22,32,33]	
		Bachelotiaceae	*Bachelotia* (Bornet) Kukuck ex Hamel	*B. antillarum* (Grunow) Gerloff		N	Y	N	N	N	[14]	
	Sphacelariales	Stypocaulaceae	*Halopteris* Kützing	*H. filicina* (Grateloup) Kützing		N	Y	N	N	N	[14]	
		Sphacelariaceae	*Sphacelaria* Lyngbye	*S. novae-hollandiae* Sonder		N	Y	N	N	N	[9,14,22]	
				*S. rigidula* Kützing	*S. furcigera* Kützing	N	Y	N	N	N	[9,14,22]	
				*S. tribuloides* Meneghini		N	Y	Y	N	N	[9,14,22,32,33]	
	Sporochnales	Sporochnaceae	*Nereia* Zanardini	*N. intricata* Yamada		N	Y	N	N	N	[14,22]	
			*Sporochnus* C.Agardh	*S. dotyi* Brostoff		N	Y	N	N	N	[14,22]	
				*S. moorei* Harvey		N	Y	N	N	N	[14,22]	
	Tilopteridales	Cutleriaceae	*Cutleria* Greville	*C. irregularis* I.A.Abbott and Huisman		N	Y	N	N	N	[14]	
Rhodophyta	Acrochaetiales	Acrochaetiaceae	*Acrochaetium* Nägeli	*A. actinocladum* I.A.Abbott		Y (?)	Y	N	N	N	[12]	
				*A. barbadense* (Vickers) Børgesen	*Audouinella barbadensis* (Vickers) Woelkerling	N	Y	N	N	N	[12]	
				*A. crassipes* (Børgesen) Børgesen		N	Y	N	N	N	[38]	
				*A. dotyi* I.A.Abbott		Y	Y	N	N	N	[12]	
				*A. imitator* I.A.Abbott		Y (?)	Y	N	N	N	[12]	
				*A. liagorae* Børgesen	*Audouinella liagorae* (Børgesen) Woelkerling	N	Y	N	N	N	[12]	
				*A. microscopicum* (Nägeli ex Kützing) Nägeli		N	Y	N	N	N	[9,12,22]	
				*A. trichogloeae* Børgesen		N	Y	N	N	N	[12]	
			*Liagorophila* Yamada	*L. endophytica* Yamada		N	Y	N	N	N	[12]	
	Acrosymphytales	Acrosymphytaceae	*Acrosymphyton* Sjöstedt	*A. brainardii* Vroom and I.A.Abbott		Y	Y	N	N	N	[22,39]	
				*A. taylorii* I.A.Abbott		N	Y	Y	Y	N	[12,24]	
	Bangiales	Bangiaceae	*Bangia* Lyngbye	*B. fuscopurpurea* (Dillwyn) Lyngbye	*B. atropurpurea* (Mertens ex Roth) C.Agardh	N	Y	Y	N	N	[12,22,24]	
			*Pyropia* J.Agardh	*P. acanthophora* (E.C.Oliveira and Coll) Santiañez	*Porphyra acanthophora* E.C.Oliveira and Coll	N	Y	Y	Y	N	[40]	
				*P. vietnamensis* (Tak.Tanaka and P.H.Hô) J.E.Sutherland and Monotilla	*Phycocalidia vietnamensis* (Tak.Tanaka and P.H.Hô) Santiañez and M.J.Wynne, *Porphyra vietnamensis* Tak.Tanaka and P.H.Hô	N	Y	Y	N	N	[9,12,22,24]	
			*Pseudobangia* K.M.Müller and R.G.Sheath	*Pseudobangia* sp.		U	Y	Y	N	N	[24]	
	Bonnemaisoniales	Bonnemaisoniaceae	*Asparagopsis* Montagne	*A.taxiformis* (Delile) Trevisan		N	Y	Y	Y	N	[9,12,22,41,42]	
		Naccariaceae	*Naccaria* Endlicher	*N. hawaiiana* I.A.Abbott		Y	Y	N	N	N	[12]	
			*Reticulocaulis* I.A.Abbott	*R. mucosissimus* I.A.Abbott		N	Y	Y	N	N	[9,12,24]	
	Ceramiales	Callithamniaceae	*Aglaothamnion* Feldmann-Mazoyer	*A. boergesenii* (Aponte and D.L.Ballantine) L’Hardy-Halos and Rueness		N	Y	Y	N	N	[12,22,24]	
				*A. cordatum* (Børgesen) Feldmann-Mazoyer	*Callithamnion neglectum* (Feldmann-Mazoyer) M.J.Wynne	N	Y	Y	N	N	[12,22,24]	
			*Crouania* J.Agardh	*C. attenuata* (C.Agardh) J.Agardh		N	Y	N	N	N	[22,43,44]	
				*C. mageshimensis* Itono		N	Y	N	N	N	[9,12,22,45]	
				*C. minutissima* Yamada		N	Y	N	N	N	[9,12,24]	
			*Euptilocladia* Wollaston	*E. magruderi* I.A.Abbott and R.E.Norris		Y	Y	Y	Y	N	[9,12,24]	
			*Gymnothamnion* J.Agardh	*G. elegans* (Schousboe ex C.Agardh) J.Agardh		N	Y	Y	N	N	[9,12,24]	
			*Ptilocladia* Sonder	*P. yuenii* I.A.Abbott		N	Y	N	N	N	[12,22]	
			*Spyridia* Harvey	*S. filamentosa* (Wulfen) Harvey		N	Y	Y	N	N	[9,12,22,46]	
		Ceramiaceae	*Acrothamnion* J.Agardh	*A. butlerae* (Collins) Kylin		N	Y	Y	N	N	[12,22]	
			*Antithamnion* Nägeli	*A. antillanum* Børgesen		N	Y	Y	N	N	[12,22,24]	
				*A. decipiens* (J.Agardh) Athanasiadis	*A. ogdeniae* I.A.Abbott	N	Y	Y	N	N	[12,22,24,47]	
				*A. erucacladellum* R.E.Norris		Y	Y	Y	N	N	[12,24]	
				*A. nipponicum* Yamada and Inagaki		N	Y	N	N	N	[12]	
			*Antithamnionella* Lyle	*A. breviramosa* (E.Y.Dawson) Wollaston		N	Y	N	N	N	[12,22]	
				*A. graeffei* (Grunow) Athanasiadis		N	Y	N	N	N	[12,22]	
			*Ardreanema* R.E.Norris and I.A.Abbott	*A. seriosporum* (E.Y.Dawson) R.E.Norris		N	Y	N	N	N	[12]	
			*Balliella* Itono and Tak.Tanaka	*B. repens* Huisman and Kraft		N	Y	Y	N	N	[12,24]	
			*Callidictyon* J.N.Norris and I.A.Abbott	*C. abyssorum* J.N.Norris and I.A.Abbott		Y	Y	N	N	N	[12]	
			*Callithamniella* Feldmann-Mazoyer	*C. pacifica* I.A.Abbott and R.E.Norris		N	Y	N	N	N	[12]	
			*Centroceras* Kützing	*C. clavulatum* (C.Agardh) Montagne		N	Y	Y	N	N	[9,12,22,24]	
				*C. corallophiloides* R.E.Norris		Y	Y	N	N	N	[9,12,22,24]	
				*C. gasparrinii* (Meneghini) Kützing		N	Y	Y	N	N	[48]	
				*C. minutum* Yamada		N	Y	N	N	N	[9,12,22]	
			*Ceramium* Roth	*C. aduncum* Nakamura		N	Y	N	N	N	[12,22]	
				*C. affine* Setchell and N.L.Gardner		N	Y	N	N	N	[22]	
				*C. borneense* Weber Bosse		N	Y	N	N	N	[12,22]	
				*C. cingulum* Meneses		Y	Y	N	N	N	[12,22]	
				*C. clarionense* Setchell and N.L.Gardner		N	Y	N	N	N	[9,12,22]	
				*C. codii* (H.Richards) Mazoyer		N	Y	N	N	N	[9,12,22]	
				*C. diaphanum* (Lightfoot) Roth	*C. tenuissimum* (Roth) Areschoug	N	Y	N	N	N	[12,22]	
				*C. dumosertum* R.E.Norris and Abbott		N	Y	Y	Y	N	[9,12,22,24]	
				*C. hamatispinum* E.Y.Dawson		N	Y	N	N	N	[12,22]	
				*C. jolyi* (Díaz-Piferrer) D.L.Ballantine and M.J.Wynne		N	Y	N	N	N	[12]	
				*C. macilentum* J.Agardh	*C. mazatlanense* E.Y.Dawson	N	Y	N	N	N	[12,22]	
				*C. nakamurae* E.Y.Dawson	*C. hanaense* R.E.Norris and I.A.Abbott	N	Y	N	N	N	[12]	
				*C. paniculatum* Okamura		N	Y	N	N	N	[12]	
				*C. punctiforme* Setchell		N	Y	N	N	N	[12]	
				*C. serpens* Setchell and N.L.Gardner		N	Y	N	N	N	[12]	
				*C. tranquillum* Meneses		Y	Y	N	N	N	[12]	
				*C. vagans* P.C.Silva		N	Y	N	N	N	[12,22]	
				*C. womersleyi* R.E.Norris and I.A.Abbott		Y	Y	Y	Y	N	[12,22,24]	
			*Corallophila* Weber Bosse	*C. huysmansii* (Weber Bosse) R.E.Norris		N	Y	N	N	N	[12,22]	
				*C. itonoi* (Ardré) R.E.Norris		N	Y	N	N	N	[12]	
				*C. kleiwegii* Weber Bosse	*C. apiculata* (Yamada) R.E.Norris	N	Y	N	N	N	[12,22]	
				*C. ptilocladioides* (R.E.Norris and I.A.Abbott) R.E.Norris		Y	Y	N	N	N	[12]	
			*Delesseriopsis* Okamura	*D. elegans* Okamura		N	Y	N	N	N	[12]	
			*Gayliella* T.O.Cho, L.M.McIvor and S.M.Boo	*G. fimbriata* (Setchell and N.L.Gardner) T.O.Cho and S.M.Boo	*Ceramium fimbriatum* Setchell and N.L.Gardner	N	Y	Y	Y	N	[9,12,22,24]	
				*G. flaccida* (Harvey ex Kützing) T.O.Cho and L.J.McIvor	*Ceramium flaccidum* (Harvey ex Kützing) Ardissone	N	Y	N	N	N	[9,12,22]	
			*Ossiella* A.J.K.Millar and I.A.Abbott	*O. pacifica* A.J.K.Millar and I.A.Abbott		N	Y	N	N	N	[12]	
			*Perikladosporon* Athanasiadis	*P. percurrens* (E.Y.Dawson) Athanasiadis	*Antithamnion percurrens* E.Y.Dawson	N	Y	Y	N	N	[12,24]	
		Delesseriaceae	*Branchioglossum* Kylin	*B. prostratum* C.W.Schneider		N	Y	N	N	N	[12,22]	
			*Cryptopleura* Kützing	*C. peltata* (Montagne) M.J.Wynne	*C. corallinarum* (Nott) N.L.Gardner	N	Y	N	N	N	[12]	
			*Dasya* C.Agardh	*D. anastomosans* (Weber Bosse) M.J.Wynne	*D. pilosa* (Weber Bosse) A.J.K.Millar	N	Y	Y	N	N	[12,22,24]	
				*D. atropurpurea* Vroom		Y	Y	N	N	N	[22,49]	
				*D. pedicellata* (C.Agardh) C.Agardh	*D. baillouviana* (S.G.Gmelin) Montagne	N	Y	Y	N	N	[22,24]	Doubtful record. Detailed analyses may demonstrate this to be an undescribed species [50].
				*D. corymbifera* J.Agardh		N	Y	Y	N	N	[9,12,22,24]	
				*D. iridescens* (Schlech) A.J.K.Millar and I.A.Abbott	*Eupogodon iridescens* Schlech	N	Y	Y	Y	N	[9,12,22,24]	
				*D. kristeniae* I.A.Abbott		N	Y	Y	N	N	[12,22,24]	
				*D. murrayana* I.A.Abbott and A.J.K.Millar		N	Y	Y	N	N	[12,24]	
				*D. villosa* Harvey		N	Y	N	N	N	[22]	
			*Dotyella* Womersley and Shepley	*D. hawaiiensis* (Doty and Wainwright) Womersley and Shepley		N	Y	N	N	N	[9,12,22]	
				*D. hawaiiensis* var. *clavata* Hollenberg		Y	Y	N	N	N	[51]	
				*D. irregularis* I.A.Abbott		N	Y	N	N	N	[9,12,22]	
			*Haraldiophyllum* A.D.Zinova	*H. hawaiiense* M.O.Paiano, Huisman and A.R.Sherwood		Y	Y	Y	Y	Y	[5]	
			*Heterosiphonia* Montagne	*H. crispella* (C.Agardh) M.J.Wynne	*H. wurdemannii* (Bailey ex Harvey) Falkenberg	N	Y	Y	N	N	[12,22,24,47]	
			*Hypoglossum* Kützing	*H. barbatum* Okamura		N	Y	N	N	N	[12,22]	
				*H. caloglossoides* M.J.Wynne and Kraft		N	Y	N	N	N	[12,22]	
				*H. minimum* Yamada		N	Y	N	N	N	[12,22]	
				*H. rhizophorum* D.L.Ballantine and M.J.Wynne		N	Y	Y	N	N	[12,22,24]	
				*H. simulans* M.J.Wynne, I.R.Price and D.L.Ballantine		N	Y	N	N	N	[12,22]	
				*H. wynnei* I.A.Abbott		Y	Y	N	N	N	[12]	
			*Malaconema* Womersley and Shepley	*M. minimum* Hollenberg		N	Y	Y	N	N	[12,22,24]	
			*Martensia* K.Hering	*M. abbottiae* A.R.Sherwood and S.-M.Lin		Y	Y	Y	Y	Y	[6]	
				*M. albida* Y.Lee	*M. fragilis* Harvey, *M. flabelliformis* Harvey ex J.Agardh	N	Y	Y	Y	N	[6,9,12,22]	Many records of *M. fragilis* and *M. flabelliformis* in Hawai‘i correspond to this species [6].
				*M. hawaiiensis* A.R.Sherwood and S.-M.Lin	*M. flabelliformis* Harvey ex J.Agardh	Y	Y	Y	Y	Y	[6,9,12,22]	Many records of *M. flabelliformis* in Hawai‘i correspond to this species [6].
				*M. lauhiekoeloa* A.R.Sherwood and S.-M.Lin		Y	Y	Y	Y	Y	[6]	
				*M. tsudae* A.R.Sherwood and S.-M.Lin	*M. fragilis* Harvey	Y	Y	Y	Y	Y	[6,9,12,22]	Many records of *M. fragilis* in Hawai‘i correspond to this species [6].
			*Nitophyllum* Greville	*N. adhaerens* M.J.Wynne		N	Y	Y	N	N	[12,22,24]	
			*Phrix* J.G.Stewart	*P. spatulata* (E.Y.Dawson) M.J.Wynne, M.Kamiya and J.A.West	*Apoglossum gregarium* (E.Y.Dawson) M.J.Wynne	N	Y	N	N	N	[12]	
			*Schizoseris* Kylin	*S. bombayensis* (Børgesen) Showe M.Lin	*Myriogramme bombayensis* Børgesen	N	Y	N	N	N	[12,22]	
			*Taenioma* J.Agardh	*T. perpusillum* (J.Agardh) J.Agardh		N	Y	Y	N	N	[12,22,24]	
			*Vanvoorstia* Harvey	*V. coccinea* Harvey ex J.Agardh		N	Y	Y	N	N	[12,22,24]	
				*V. spectabilis* Harvey		N	Y	N	N	N	[12,22]	
		Rhodomelaceae	*Acanthophora* J.V.Lamouroux	*A. pacifica* (Setchell) Kraft	*Cladhymenia pacifica* Setchell	N	Y	Y	N	N	[9,12]	
				*A. spicifera* (M.Vahl) Børgesen		N	Y	Y	N	N	[9,12,24]	
			*Amansia* J.V.Lamouroux	*A. fimbrifolia* (R.E.Norris) L.E.Phillips	*Melanamansia fimbrifolia* R.E.Norris	N	Y	Y	N	N	[12,22,24,52]	
				*A. glomerata* C.Agardh	*Melanamansia glomerata* (C.Agardh) R.E.Norris	N	Y	Y	Y	Y	[9,12,22,24,52]	*A. daemelii* (Sonder) J.Agardh should no longer be considered part of the Hawaiian flora; these specimens represent *A. glomerata* [52]
			*Chondria* C.Agardh	*C. arcuata* Hollenberg		N	Y	Y	N	N	[12,19,22,24]	
				*C. dangeardii* E.Y.Dawson		N	Y	Y	N	N	[12,19,22,24,53]	
				*C. minutula* Weber Bosse		N	Y	N	N	N	[9,12,22]	
				*C. polyrhiza* Collins and Hervey		N	Y	N	N	N	[9,12,22]	
				*C. simpliciuscula* Weber Bosse		N	Y	N	N	N	[12,22]	
				*C. tumulosa* A.R.Sherwood and Huisman		Y	Y	Y	Y	Y	[19]	
			*Chondrophycus* (J.Tokida and Y.Saito) Garbary and J.T.Harper	*C. cartilagineus* (Yamada) Garbary and J.T.Harper	*Laurencia cartilaginea* Yamada	N	Y	Y	N	N	[9,12,22,24]	
				*C. dotyi* (Y.Saito) K.W.Nam	*Laurencia dotyi* Saito	N	Y	Y	Y	N	[9,12,22,24]	
				*C. succisus* (A.B.Cribb) K.W.Nam	*Laurencia succisa* A.B.Cribb	N	Y	Y	N	N	[9,12,24,53]	
				*C. undulatus* (Yamada) Garbary and Harper	*Laurencia undulata* Yamada	N	Y	Y	N	N	[12,24]	
			*Digenea* C.Agardh	*D. cymatophila* (R.E.Norris) Díaz-Tapia and Maggs	*Alsidium cymatophilum* R.E.Norris	N	Y	Y	Y	N	[12,24]	
			*Ditria* Hollenberg	*D. reptans* Hollenberg		N	Y	N	N	N	[12,22]	
			*Epizonaria* Díaz-Tapia and Maggs	*E. prostrata* (Harvey) Díaz-Tapia and Maggs	*Lophosiphonia prostrata* (Harvey) Falkenberg	N	Y	N	N	N	[12,22]	
			*Exophyllum* Weber Bosse	*E. wentii* Weber Bosse		N	Y	N	N	N	[12]	
			*Hawaiia* Hollenberg	*H. trichia* Hollenberg		Y	Y	N	N	N	[12]	
			*Herposiphonia* Nägeli	*H. arcuata* Hollenberg		N	Y	N	N	N	[12,54]	
				*H. crassa* Hollenberg		N	Y	N	N	N	[12,54]	
				*H. delicatula* Hollenberg		N	Y	N	N	N	[12,22,54]	
				*H. dendroidea* Hollenberg		N	Y	N	N	N	[22,54]	
				*H. dubia* Hollenberg		N	Y	N	N	N	[12,22,54]	
				*H. nuda* Hollenberg		N	Y	N	N	N	[12,22,54]	
				*H. obscura* Hollenberg		N	Y	N	N	N	[12,22,54]	
				*H. pacifica* Hollenberg		N	Y	N	N	N	[9,12,22,54]	
				*H. parca* Setchell		N	Y	N	N	N	[9,12,22,54]	
				*H. secunda* (C.Agardh) Ambronn		N	Y	N	N	N	[9,12,22]	
				*H. tenella* (C.Agardh) Ambronn		N	Y	N	N	N	[22,54]	
				*H. variabilis* Hollenberg		N	Y	N	N	N	[12,22,54]	
			*Janczewskia* Solms-Laubach	*J. hawaiiana* Apt		Y	Y	Y	Y	N	[12,24,53]	
			*Kapraunia* Savoie and G.W.Saunders	*K. pentamera* (Hollenberg) Savoie and G.W.Saunders	*Polysiphonia pentamera* Hollenberg	N	Y	N	N	N	[12]	
			*Laurencia* J.V.Lamouroux	*L. brachyclados* Pilger		N	Y	Y	N	N	[9,12,22,24,55]	
				*L. decumbens* Kützing		N	Y	Y	N	N	[12,22,24]	
				*L. dendroidea* J.Agardh	*L. majuscula* (Harvey) A.H.S.Lucas	N	Y	Y	N	N	[9,12,22,24,55]	
				*L. elegans* A.H.S.Lucas		N	Y	Y	Y	N	[55]	
				*L. galtsoffii* M.Howe		N	Y	Y	N	N	[12,22,24]	
				*L. glandulifera* (Kützing) Kützing		N	Y	N	N	N	[12]	
				*L. mariannensis* Yamada		N	Y	N	N	N	[12,22]	
				*L. mcdermidiae* I.A.Abbott		N	Y	Y	Y	N	[9,12,24,55]	
				*L. nidifica* J.Agardh		N	Y	Y	Y	N	[12,22,24,55]	
				*L. obtusa* (Hudson) J.V.Lamouroux		N	Y	N	N	N	[13,44]	
				*L. subsimplex* C.K.Tseng		N	Y	N	N	N	[22]	
				*L. tenera* C.K.Tseng		N	Y	N	N	N	[12]	
			*Leveillea* Decaisne	*L. jungermannioides* (Hering and G.Martens) Harvey		N	Y	Y	N	N	[12,24]	
			*Lophocladia* (J.Agardh) F.Schmitz	*L. kipukaia* Schlech		N	Y	N	N	N	[12]	
				*L. kuesteri* I.A.Abbott, D.L.Ballantine and O’Doherty		Y	Y	Y	Y	N	[22,56]	
				*L. trichoclados* (C.Agardh) F.Schmitz		N	Y	N	N	N	[22]	
			*Lophosiphonia* Falkenberg	*L. cristata* Falkenberg		N	Y	N	N	N	[12,22]	
			*Melanothamnus* Bornet and Falkenberg	*M. apiculatus* (Hollenberg) Díaz- Tapia and Maggs	*Polysiphonia apiculata* Hollenberg, *Neosiphonia apiculata* (Hollenberg) Masuda and Kogame	N	Y	N	N	N	[12,22,57]	
				*M. delicatulus* (Hollenberg) Huisman	*Polysiphonia delicatula* Hollenberg	N	Y	N	N	N	[12,22,57]	
				*M. ecorticatus* (R.E.Norris) Díaz-Tapia and Maggs	*Fernandosiphonia ecorticata* R.E.Norris	N	Y	N	N	N	[12]	
				*M. hancockii* (E.Y.Dawson) Díaz-Tapia and Maggs	*Polysiphonia hancockii* E.Y.Dawson	N	Y	N	N	N	[12]	
				*M. hawaiiensis* (Hollenberg) Díaz-Tapia and Maggs	*Neosiphonia hawaiiensis* (Hollenberg) M.-S.Kim and I.A.Abbott, *Polysiphonia hawaiiensis* Hollenberg	N	Y	N	N	N	[9,12,57]	
				*M. nanus* (A.J.K.Millar) Díaz-Tapia and Maggs	*Fernandosiphonia nana* A.J.K.Millar	N	Y	N	N	N	[12]	
				*M. polyphysus* (Kützing) Díaz-Tapia and Maggs	*Neosiphonia polyphysa* (Kützing) Skelton and South, *Polysiphonia polyphysa* Kützing	N	Y	N	N	N	[22]	
				*M. pseudovillum* (Hollenberg) Díaz-Tapia and Maggs	*Polysiphonia pseudovillum* Hollenberg	N	Y	N	N	N	[9,12,22,57]	
				*M. quadratus* (Hollenberg) Huisman	*Polysiphonia quadrata* Hollenberg	N	Y	N	N	N	[57,58]	
				*M. savatieri* (Hariot) Díaz-Tapia and Maggs	*Polysiphonia savatieri* Hariot, *Polysiphonia japonica* var. *savatieri* (Hariot) H.Y.Yoon, *Neosiphonia savatieri* (Hariot) M.S.Kim and I.K.Lee	N	Y	N	N	N	[12,22,57]	
				*M. simplex* (Hollenberg) Díaz-Tapia and Maggs	*Polysiphonia simplex* Hollenberg	N	Y	N	N	N	[12,22]	
				*M. sparsus* (Setchell) Díaz-Tapia and Maggs	*Polysiphonia sparsa* (Setchell) Hollenberg	N	Y	N	N	N	[12,22,57]	
				*M. sphaerocarpus* (Børgesen) Díaz-Tapia and Maggs	*Neosiphonia sphaerocarpa* (Børgesen) M.-S.Kim and I.K.Lee, *Polysiphonia sphaerocarpa* Børgesen	N	Y	N	N	N	[9,12,22,57]	
				*M. tongatensis* (Harvey ex Kützing) Díaz-Tapia and Maggs	*Polysiphonia tongatensis* Harvey ex Kützing	N	Y	N	N	N	[12]	
				*M. upolensis* (Grunow) Díaz-Tapia and Maggs	*Neosiphonia upolensis* (Grunow) M.S.Kim and Boo, *Polysiphonia upolensis* Grunow	N	Y	Y	N	N	[9,12,22,24,57]	
			*Micropeuce* J.Agardh	*M. setosa* I.A.Abbott		Y	Y	N	N	N	[12]	
			*Neotenophycus* Kraft and I.A.Abbott	*N. ichthyosteus* Kraft and I.A.Abbott		N	Y	N	N	N	[59]	
			*Osmundaria* J.V.Lamouroux	*O. fimbriata* (J.V.Lamouroux) R.E.Norris	*Vidalia fimbriata* (Lamouroux) J.Agardh	N	Y	N	N	N	[58]	
				*O. obtusiloba* (C.Agardh) R.E.Norris		N	Y	Y	N	N	[12,24]	
			*Palisada* K.W.Nam	*P. crustiformans* (K.J.McDermid) A.R.Sherwood, A.Kurihara and K.W.Nam	*Laurencia crustiformans* K.J.McDermid	N	Y	Y	Y	N	[9,12,22,24,60]	
				*P. parvipapillata* (C.K.Tseng) K.W.Nam	*Laurencia parvipapillata* C.K.Tseng, *Chondrophycus parvipapillatus* (C.K.Tseng) Garbary and J.T.Harper	N	Y	Y	N	N	[9,12,22,24]	
				*P. perforata* (Bory) K.W.Nam		N	Y	N	N	N	[22]	
				*P. surculigera* (C.K.Tseng) K.W.Nam		N	Y	N	N	N	[22]	
				*P. thuyoides* (Kützing) Cassano, Sentíes, Gil-Rodríguez and M.T.Fujii	*Laurencia paniculata* (C.Agardh) J.Agardh	N	Y	N	N	N	[61]	Doubtful record.
				*P. yamadana* (M.Howe) K.W.Nam	*Laurencia yamadana* M.Howe	N	Y	Y	N	N	[12,22,24]	
			*Phaeocolax* Hollenberg	*P. kajimurae* Hollenberg		N	Y	N	N	N	[12]	
			*Polysiphonia* Greville	*P. anomala* Hollenberg		N	Y	N	N	N	[12,57]	
				*P. beaudettei* Hollenberg	*Neosiphonia beaudettei* (Hollenberg) M.S.Kim and I.A.Abbott	N	Y	N	N	N	[12,22]	
				*P. exilis* Harvey		N	Y	N	N	N	[12,22]	
				*P. homoia* Setchell and N.L.Gardner		N	Y	N	N	N	[12]	
				*P. poko* Hollenberg	*Neosiphonia poko* (Hollenberg) I.A.Abbott	N	Y	N	N	N	[9,12,22,57]	
				*P. profunda* Hollenberg	*Neosiphonia profunda* (Hollenberg) M.-S.Kim and I.A.Abbott	Y	Y	N	N	N	[12,57]	
				*P. rubrorhiza* Hollenberg	*Neosiphonia rubrorhiza* (Hollenberg) M.-S.Kim and I.A.Abbott	Y	Y	N	N	N	[12,22,57]	
				*P. saccorhiza* (Collins and Hervey) Hollenberg		N	Y	N	N	N	[12,22,57]	
				*P. scopulorum* Harvey		N	Y	N	N	N	[12,22,57]	
				*P. sertularioides* (Grateloup) J.Agardh	*Neosiphonia sertularioides* (Grateloup) K.W.Nam and P.J.Kang, *P. flaccidissima* Hollenberg	N	Y	N	N	N	[12,22,57]	
				*P. subtilissima* Montagne		N	Y	Y	N	N	[12,57,62]	
				*P. triton* P.C.Silva		N	Y	N	N	N	[12]	
				*P. tsudana* Hollenberg		N	Y	N	N	N	[12,22]	
				*P. tuberosa* Hollenberg		Y	Y	N	N	N	[12,22,57]	
				*P. villum* J.Agardh		N	Y	N	N	N	[38]	
			*Rhodolachne* M.J.Wynne	*R. decussata* M.J.Wynne		N	Y	Y	N	N	[12,24]	
			*Spirocladia* Børgesen	*S. barodensis* Børgesen		N	Y	Y	N	N	[9,12,24]	
				*S. hodgsoniae* I.A.Abbott		Y	Y	Y	Y	N	[9,12,22,24]	
			*Symphyocladia* Falkenberg	*S. marchantioides* (Harvey) Falkenberg		N	Y	N	N	N	[12]	
			*Tayloriella* Kylin	*T. dictyurus* (J.Agardh) Kylin		N	Y	Y	N	N	[12,24]	
			*Tolypiocladia* F.Schmitz	*T. glomerulata* (C.Agardh) F.Schmitz		N	Y	Y	N	N	[9,12,22,24]	
			*Ululania* K.E.Apt and K.E.Schlech	*U. stellata* K.E.Apt and K.E.Schlech		Y	Y	Y	Y	N	[12,24,53]	
			*Vertebrata* S.F.Gray	*V. foetidissima* (Cocks ex Bornet) Díaz-Tapia and Maggs	*Polysiphonia tepida* Hollenberg, *P. foetidissima* Cocks ex Bornet, *Neosiphonia tepida* (Hollenberg) S.M.Guimarães and M.T.Fujii	N	Y	Y	N	N	[12,22,24]	
			*Wilsonosiphonia* D.Bustamante, Won and T.O.Cho	*W. howei* (Hollenberg) D.Bustamante, Won and T.O.Cho	*Polysiphonia howei* Hollenberg	N	Y	Y	N	N	[9,12,24]	
			*Womersleyella* Hollenberg	*W. herpa* (Hollenberg) R.E.Norris	*Polysiphonia herpa* Hollenberg	N	Y	N	N	N	[12,22,57]	
				*W. pacifica* Hollenberg		N	Y	N	N	N	[12,22]	
				*W. setacea* (Hollenberg) R.E.Norris	*Polysiphonia setacea* Hollenberg	N	Y	N	N	N	[12,57]	
			*Xiphosiphonia* Savoie and G.W.Saunders	*X. pennata* (C.Agardh) Savoie and G.W.Saunders	*Pterosiphonia pennata* (C.Agardh) Sauvageau	N	Y	N	N	N	[12]	
		Wrangeliaceae	*Anotrichium* Nägeli	*A. secundum* (Harvey ex J.Agardh) G.Furnari		N	Y	N	N	N	[9,12,22]	
				*A. tenue* (C.Agardh) Nägeli		N	Y	Y	N	N	[9,12,22,24]	
			*Diplothamnion* A.B.Joly and Yamaguishi	*D. jolyi* C.Hoek		N	Y	Y	N	N	[12,22,24]	
			*Griffithsia* C.Agardh	*G. heteromorpha* Kützing		N	Y	Y	N	N	[9,12,22,24]	
				*G. metcalfii* C.K.Tseng		N	Y	N	N	N	[12]	
				*G. schousboei* Montagne		N	Y	Y	N	N	[12,22,24]	
				*G. subcylindrica* Okamura		N	Y	Y	N	N	[9,12,24]	
			*Haloplegma* Montagne	*H. duperreyi* Montagne		N	Y	Y	N	N	[9,12,22,24]	
			*Lejolisia* Bornet	*L. pacifica* Itono		N	Y	Y	N	N	[12,22,24]	
			*Monosporus* Solier	*M. indicus* Børgesen		N	Y	Y	N	N	[12,24]	
			*Pleonosporium* Nägeli	*P. intricatum* R.E.Norris		Y	Y	N	N	N	[12]	
			*Ptilothamnion* Thuret	*P. cladophorae* (Yamada and T.Tanaka) G.Feldmann-Mazoyer		N	Y	N	N	N	[12,22]	
			*Spongoclonium* Sonder	*S. caribaeum* (Børgesen) M.J.Wynne	*Pleonosporium caribaeum* (Børgesen) R.E.Norris	N	Y	N	N	N	[12]	
			*Tiffaniella* Doty and Meñez	*T. saccorhiza* (Setchell and N.L.Gardner) Doty and Meñez		N	Y	N	N	N	[12,22]	
			*Wrangelia* C.Agardh	*W. dumontii* (E.Y.Dawson) I.A.Abbott		N	Y	N	N	N	[9,12,22]	
				*W. elegantissima* R.E.Norris		N	Y	Y	Y	N	[9,12,22,24]	
				*W. penicillata* (C.Agardh) C.Agardh		N	Y	N	N	N	[13]	
	Colaconematales	Colaconemataceae	*Colaconema* Batters	*C. corymbiferum* (Thuret) Alongi, Cormaci and G.Furnari	*Acrochaetium corymbiferum* (Thuret) Batters	N	Y	N	N	N	[12]	
				*C. hypneae* (Børgesen) A.A.Santos and C.W.N.Moura	*Acrochaetium hypneae* (Børgesen) Børgesen, *A. seriatum* Børgesen	N	Y	N	N	N	[9]	
				*C. nemalii* (De Notaris ex Dufour) Stegenga	*Acrochaetium nemalii* (De Notaris ex Dufour) Børgesen	N	Y	N	N	N	[12]	Abbott [12] lists “*A. nemalionis* (DeNotaris ex Ardissone) Bornet”; the assumption in AlgaeBase is that she meant *A. nemalii* (DeNotaris ex Dufour) Børgesen. There is no taxon by the former name listed in AlgaeBase.
				*C. robustum* (Børgesen) Huisman and Woelkerling	*Acrochaetium robustum* Børgesen	N	Y	N	N	N	[12]	
	Corallinales	Corallinaceae	*Arthrocardia* Decaisne	*Arthrocardia* sp.		U	Y	Y	N	N	[24]	
			*Ellisolandia* K.R.Hind and G.W.Saunders	*E. elongata* (J.Ellis and Solander) K.R.Hind and G.W.Saunders	*Corallina elongata* J.Ellis and Solander	N	Y	N	N	N	[12]	
			*Jania* J.V.Lamouroux	*J. micrarthrodia* J.V.Lamouroux		N	Y	N	N	N	[9,12,22]	
				*J. pedunculata* var. *adhaerens* (J.V.Lamouroux) A.S.Harvey, Woelkerling and Reviers	*J. adhaerens* J.V.Lamouroux	N	Y	N	N	N	[12,22]	
				*J. pumila* J.V.Lamouroux		N	Y	N	N	N	[9,12,22]	
				*J. subulata* (Ellis and Solander) Sonder	*Haliptilon subulatum* (J.Ellis and Solander) H.W.Johansen	N	Y	Y	N	N	[9,12,22,24]	
				*J. tenella* (Kützing) Grunow		N	Y	N	N	N	[47]	
				*J. verrucosa* J.V.Lamouroux nom. rejic.		N	Y	N	N	N	[12,22]	The status of *Jania* species in Hawai‘i requires intensive molecular study.
		Hapalidiaceae	*Choreonema* F.Schmitz	*C. thuretii* (Bornet) F.Schmitz		N	Y	N	N	N	[22,44]	
			*Phymatolithon* Foslie	*Phymatolithon* sp.		U	Y	Y	N	N	[24]	
		Hydrolithaceae	*Hydrolithon* (Foslie) Foslie	*H. boergesenii* (Foslie) Foslie	*H. reinboldii* (Weber Bosse and Foslie) Foslie	N	Y	Y	N	N	[22,24]	
				*H. breviclavium* (Foslie) Foslie		N	Y	N	N	N	[13,63]	
				*H. farinosum* (J.V.Lamouroux) Penrose and Y.M.Chamberlain		N	Y	N	N	N	[9,22]	
		Lithophyllaceae	*Amphiroa* J.V.Lamouroux	*A. beauvoisii* J.V.Lamouroux		N	Y	Y	N	N	[9,12,22]	
				*A. foliacea* J.V.Lamouroux		N	Y	Y	N	N	[12,24]	
				*A. fragilissima* (Linnaeus) J.V.Lamouroux		N	Y	N	N	N	[13,22]	
				*A. rigida* J.V.Lamouroux		N	Y	N	N	N	[9,12,22]	
				*A. valonioides* Yendo		N	Y	Y	N	N	[12,22,24]	
			*Lithophyllum* Philippi	*L. ganeopsis* W.H.Adey, R.A.Townsend and Boykins		Y	Y	N	N	N	[38,64]	
				*L. insipidum* W.H.Adey, R.A.Townsend and Boykins		N	Y	Y	Y	N	[24,65]	
				*L. kotschyanum* Unger		N	Y	Y	N	N	[9,13,24,65]	
				*L. prototypum* (Foslie) Foslie	*Tenarea tessellata* (Lemoine) Littler ex Adey et al., *Titanoderma tessellatum* (Me.Lemoine) Woelkerling, Y.M.Chamberlain and P.C.Silva	N	Y	N	N	N	[66,67]	Doubtful record
				*L. subreduncum* Foslie		Y	Y	Y	Y	Y	[68,69]	According to Basso et al. [69], known only from the type locality and other reports (from AlgaeBase) require verification.
		Mastophoraceae	*Mastophora* Decaisne	*M. pacifica* (Heydrich) Foslie		N	Y	N	N	N	[9,70]	
				*M. rosea* (C.Agardh)Setchell		N	Y	N	N	N	[22,43]	
			*Metamastophora* Setchell	*Metamastophora* sp.		U	Y	Y	N	N	[24]	
		Mesophyllumaceae	*Mesophyllum* Me.Lemoine	*M. erubescens* (Foslie) Me.Lemoine		N	Y	Y	Y	N	[65,71]	
				*M. mesomorphum* (Foslie) W.H.Adey		N	Y	N	N	N	[13]	
				*M. syrphetodes* W.H.Adey, R.A.Townsend and Boykins		N	Y	N	N	N	[64]	
		Porolithaceae	*Dawsoniolithon* Caragnano, Foetisch, Maneveldt and Payri	*D. conicum* (E.Y.Dawson) Caragnano, Foetisch, Maneveldt and Payri	*Pneophyllum conicum* (E.Y.Dawson) Keats, Y.M.Chamberlain and M.Baba	N	Y	Y	N	N	[9,65]	
			*Porolithon* Foslie	*P. gardineri* (Foslie) Foslie	*Hydrolithon gardineri* (Foslie) Verheij and Prud’homme	N	Y	Y	N	N	[9,13,24,65]	
				*P. onkodes* (Heydrich) Foslie	*Hydrolithon onkodes* (Heydrich) Penrose and Woelkerling	N	Y	Y	N	N	[9,24,29,72,73]	
		Spongitidaceae	*Neogoniolithon* Setchell and L.R.Mason	*N. brassica-florida* (Harvey) Setchell and L.R.Mason	*N. frutescens* (Foslie) Setchell and L.R.Mason	N	Y	Y	N	N	[13,22,24]	
	Erythropeltales	Erythropeltales incertae sedis	*Madagascaria* J.A.West and N.Kikuchi	*Madagascaria* sp.		U	Y	Y	N	N	[24]	
		Erythotrichiaceae	*Erythrotrichia* Areschoug	*E. carnea* (Dillwyn) J.Agardh		N	Y	N	N	N	[12,22]	
			*Sahlingia* Kornmann	*S. subintegra* (Rosenvinge) Kornmann		N	Y	Y	N	N	[9,24]	
	Gelidiales	Gelidiaceae	*Gelidium* J.V.Lamouroux	*G. arenarium* Kylin		N	Y	N	N	N	[38]	
				*G. crinale* (Hare ex Turner) Gaillon		N	Y	Y	N	N	[12,24]	
				*G. pluma* Bornet ex N.H.Loomis		Y	Y	Y	Y	N	[9,12,24]	
				*G. pusillum* (Stackhouse) Le Jolis		N	Y	Y	N	N	[9,12,22,24]	
				*G. pusillum* var. *pacificum* W.R.Taylor		N	Y	N	N	N	[74]	
				*G. reediae* N.H.Loomis		Y	Y	Y	N	N	[12,24]	
				*G. reptans* (Suhr) Kylin		N	Y	N	N	N	[38]	
		Gelidiellaceae	*Gelidiella* Feldmann and G.Hamel	*G. acerosa* (Forsskål) Feldmann and Hamel		N	Y	Y	N	N	[9,12,22,24]	
				*G. fanii* S.- M.Lin		N	Y	Y	Y	N	[75,76]	
				*G. machrisiana* E.Y.Dawson		N	Y	Y	N	N	[12,24]	
			*Millerella* G.H.Boo and S.M. Boo	*M. myrioclada* (Børgesen) G.H.Boo	*Gelidiella myrioclada* (Børgesen) Feldmann and Hamel	N	Y	N	N	N	[12,22]	
			*Parviphycus* Santelices	*P. adnatus* (E.Y.Dawson) B.Santelices		N	Y	N	N	N	[77]	
				*P. antipae* (Celan) B.Santelices	*Gelidiella antipae* Celan	N	Y	N	N	N	[12]	
				*P. womersleyanus* (Kraft and I.A.Abbott) B.Santelices	*Gelidiella womersleyana* Kraft and I.A.Abbott	Y	Y	N	N	N	[12]	
		Pterocladiaceae	*Pterocladiella* B.Santelices and Hommersand	*P. bulbosa* (N.H.Loomis) Santelices	*Pterocladia bulbosa* N.H.Loomis	N	Y	N	N	N	[12]	
				*P. caerulescens* (Kützing) Santelices and Hommersand	*Pterocladia caerulescens* (Kützing) Santelices	N	Y	Y	N	N	[9,12,24]	
				*P. caloglossoides* (M.Howe) Santelices		N	Y	N	N	N	[12,22]	
				*P. capillacea* (S.G.Gmelin) Santelices and Hommersand	*Pterocladia capillacea* (S.G.Gmelin) Bornet	N	Y	Y	N	N	[9,12,22]	
	Gigartinales	Caulacanthaceae	*Caulacanthus* Kützing	*C. ustulatus* (Turner) Kützing		N	Y	Y	N	N	[12,22,24]	
		Chondrymeniaceae	*Dissimularia* G.T.Kraft and G.W.Saunders	*D. dactylocarpa* G.T.Kraft and G.W.Saunders		N	Y	Y	Y	Y	[78]	
				*D. umbraticola* (E.Y.Dawson) G.T.Kraft and G.W.Saunders	*Cryptonemia umbraticola* E.Y.Dawson	N	Y	N	N	N	[9,12]	
		Cubiculosporaceae	*Cubiculosporum* Kraft	*C. koronicarpis* Kraft		N	Y	N	N	N	[12]	
		Cystocloniaceae	*Calliblepharis* Kützing	*C. saidana* (Holmes) M.Y.Yang and M.S.Kim	*Hypnea saidana* Holmes	N	Y	Y	N	N	[9]	Doubtful record.
				*C. yasutakei* M.O.Paiano and A.R.Sherwood		Y	Y	Y	Y	Y	[79]	
			*Hypnea* J.V.Lamouroux	*H. caraibica* Nauer, Cassano and M.C.Oliveira	*H. musciformis* (Wulfen) J.V.Lamouroux (in part?)	N	Y	Y	Y	Y	[9,12,80]	Nauer et al. [80] state that the alga known in Hawai‘i as *H. musciformis* is not the same as that species, and described *H. caraibica*, to which it belongs.
				*H. cervicornis* J.Agardh		N	Y	Y	N	N	[9,12]	These Hawaiian records are likely not *H. cervicornis* according to Nauer et al. [80].
				*H. chordacea* Kützing		N	Y	Y	N	N	[9,12,24]	
				*H. esperi* Bory nom. illeg.		N	Y	N	N	N	[22]	The status of *Hypnea* species in Hawai‘i requires intensive molecular study.
				*H. musciformis* (Wulfen) J.V.Lamouroux		N	Y	Y	N	N	[9,12,24]	
				*H. pannosa* J.Agardh		N	Y	Y	N	N	[12,22,24]	
				*H. spinella* (C.Agardh) Kützing		N	Y	Y	N	N	[12,22,24]	
				*H. tsudae* M.O.Paiano, F.P.Cabrera and A.R.Sherwood	*Calliblepharis saidana* (Holmes) M.Y.Yang and M.S.Kim, *H. saidana* Holmes	Y	Y	Y	Y	Y	[79]	Described to accommodate a Bishop Museum specimen labeled “*Calliblepharis saidana*” but which was shown to be a *Hypnea* instead.
				*H. valentiae* (Turner) Montagne		N	Y	Y	N	N	[12,22,24,32]	
			*Hypneocolax* Børgesen	*H. stellaris* Børgesen		N	Y	Y	N	N	[9,12,24]	
				*H. stellaris* f. *orientalis* Weber Bosse	*H. stellaris* subsp. *orientalis* (Weber Bosse) Womersley	N	Y	Y	N	N	[12,24]	
		Dicranemataceae	*Tylotus* J.Agardh	*T. laqueatus* Kraft, K.Y.Conklin and A.R. Sherwood		Y	Y	Y	Y	Y	[81]	
		Dumontiaceae	*Dudresnaya* P.Crouan and H.Crouan	*D. babbittiana* Abbott and K.J.McDermid		Y	Y	Y	Y	N	[8,9,22,82,83]	
				*D. hawaiiensis* R.K.S.Lee		N	Y	Y	Y	N	[8,9,12,22,84]	
				*D. littleri* I.A.Abbott	*D. lubrica* Littler	Y	Y	Y	Y	N	[9,12,83]	
			*Gibsmithia* Doty	*G. dotyi* Kraft and R.W.Ricker		N	Y	Y	Y	N	[9,12,22,24]	
				*G. hawaiiensis* Doty		Y	Y	Y	Y	N	[8,9,12,22,24,85]	
				*G. punonomaewa* A.R.Sherwood		Y	Y	Y	Y	Y	[8]	
		Etheliaceae	*Ethelia* Weber Bosse	*E. hawaiiensis* A.R.Sherwood		Y	Y	Y	Y	Y	[86]	
		Gigartinaceae	*Chondracanthus* Kützing	*C. acicularis* (Roth) Fredericq		N	Y	Y	N	N	[12,24]	
				*C. okamurae* I.A.Abbott		N	Y	N	N	N	[87]	
				*C. tenellus* (Harvey) Hommersand		N	Y	Y	N	N	[12,24]	
			*Chondrus* Stackhouse	*C. ocellatus* Holmes		N	Y	Y	N	N	[12,24]	Kittle et al. (unpubl.) demonstrated that this represents an undescribed species of *Chondrus*, not *C. ocellatus.*
			*Mazzaella* G.De Toni	*M. volans* (C.Agardh) Fredericq		N	Y	Y	N	N	[12,24]	
		Gloiosiphoniaceae	*Peleophycus* I.A.Abbott	*P. multiprocarpius* I.A.Abbott		N	Y	Y	Y	N	[12,24]	
		Kallymeniaceae	*Croisettea* M.J.Wynne	*C. haukoaweo* F.P.Cabrera and A.R.Sherwood		Y	Y	Y	Y	Y	[88]	
				*C. kalaukapuae* F.P.Cabrera and A.R.Sherwood		Y	Y	Y	Y	Y	[88]	
				*C. ohelouliuli* F.P.Cabrera and A.R.Sherwood		Y	Y	Y	Y	Y	[88]	
				*C. pakualapa* F.P.Cabrera and A.R.Sherwood		Y	Y	Y	Y	Y	[88]	
			*Kallymenia* J.Agardh	*K. sessilis* Okamura		N	Y	Y	N	N	[9,12,22,24]	
				*K. thompsonii* I.A.Abbott and McDermid		N	Y	Y	N	N	[9,22,24,89]	
			*Meredithia* J.Agardh	*M. hawaiiensis* F.P.Cabrera, Huisman and A.R.Sherwood		Y	Y	Y	Y	Y	[4]	
			*Psaromenia* D’Archino, W.A.Nelson and Zuccarello	*P. laulamaula* F.P.Cabrera, Huisman and A.R.Sherwood		Y	Y	Y	Y	Y	[4]	
		Phyllophoraceae	*Ahnfeltiopsis* P.C.Silva and DeCew	*A. flabelliformis* (Harvey) Masuda		N	Y	Y	N	N	[9,12,24]	
				*A. pygmaea* (J.Agardh) P.C.Silva and DeCew		N	Y	Y	N	N	[12,24]	
			*Besa* Setchell	*B. divaricata* (Holmes) M.S.Calderon and S.M.Boo	*Ahnfeltiopsis divaricata* (Holmes) Masuda	N	Y	Y	N	N	[12,24]	
			*Gymnogongrus* C.Martius	*G. durvillei* (Bory) M.S.Calderon and S.M.Boo	*Ahnfeltiopsis concinna* (J.Agardh) P.C.Silva and DeCew	N	Y	Y	Y	N	[9,12,22,24]	
		Rhizophillidaceae	*Portieria* Zanardini	*P. hornemannii* (Lyngbye) P.C.Silva	*Desmia hornemannii* Lyngbye	N	Y	Y	N	N	[9,12,22,24,90]	Leliaert et al. [90] identified 92 candidate species within *P. hornemannii* worldwide, and Hawaiian specimens were in 2 of these; thus, these lineages may eventually be described as new species.
		Solieriaceae	*Eucheuma* J.Agardh	*E. denticulatum* (N.L.Burman) Collins and Hervey		N	Y	Y	N	N	[9,12,24,91]	Type locality unknown.
			*Kappaphycus* Doty	*K. alvarezii* (Doty) L.M.Liao		N	Y	Y	Y	N	[9,12,24,91,92]	
				*K. striatus* (F.Schmitz) L.M.Liao		N	Y	Y	N	N	[9,12,24,91]	
			*Wurdemannia* Harvey	*W. miniata* (Sprengel) Feldmann and Hamel		N	Y	N	N	N	[12]	
	Gracilariales	Gracilariaceae	*Gracilaria* Greville	*G. abbottiana* M.D.Hoyle		N	Y	Y	Y	N	[12,24]	
				*G. bursa-pastoris* (S.G.Gmelin) P.C.Silva		N	Y	N	N	N	[13]	
				*G. coronopifolia* J.Agardh		N	Y	Y	Y	N	[9,12,22,24,93]	
				*G. dawsonii* Hoyle		Y	Y	Y	N	N	[9,12,24]	
				*G. dotyi* Hoyle		Y	Y	Y	Y	N	[12,24]	
			e	*G. epihippisora* Hoyle		Y	Y	Y	Y	N	[12,24]	
				*G. millardetii* (Montagne) J.Agardh		N	Y	N	N	N	[22]	
				*G. parvispora* I.A.Abbott		N	Y	Y	Y	N	[9,12,24]	
				*G. salicornia* (C.Agardh) E.Y.Dawson		N	Y	Y	Y	N	[9,12,24,93]	
				*G. tikvahiae* McLachlan		N	Y	Y	N	N	[12,24]	
			*Gracilariopsis* E.Y.Dawson	*G. lemaneiformis* (Bory) E.Y.Dawson, Acleto and Foldvik	*Gracilaria lemaneiformis* (Bory) Greville	N	Y	Y	N	N	[12,24]	
	Halymeniales	Grateloupiaceae	*Dermocorynus* P.Crouan and H.Crouan	*D. occidentalis* Hollenberg		N	Y	N	N	N	[12]	
			*Grateloupia* C.Agardh	*G. clarionensis* (Setchell and N.L.Gardner) S.Kawaguchi and H.W.Wang	*Prionitis clarionensis* (Setchell and N.L.Gardner) Kajimura	N	Y	N	N	N	[94]	
				*G. corymbifera* (I.A.Abbott) S.Kawaguchi and H.W.Wang	*Prionitis corymbifera* I.A.Abbott	Y	Y	N	N	N	[12]	
				*G. filicina* (J.V.Lamouroux) C.Agardh		N	Y	Y	N	N	[9,12,24]	
				*G. hawaiiana* E.Y.Dawson		Y	Y	Y	Y	N	[9,12,24]	
				*G. phuquocensis* Tanaka and Pham-Hoàng Hô		N	Y	Y	N	N	[9,12,24]	
		Halymeniaceae	*Carpopeltis* F.Schmitz	*C. bushiae* (Farlow) Kylin		N	Y	N	N	N	[12]	
			*Cryptonemia* J.Agardh	*C. yendoi* Weber Bosse		N	Y	Y	N	N	[9,12,24]	
			*Halymenia* C.Agardh	*H. actinophysa* M.Howe		N	Y	N	N	N	[12]	
				*H. chiangiana* I.A.Abbott and Kraft		Y	Y	N	N	N	[12]	
				*H. cromwellii* I.A.Abbott		Y	Y	N	N	N	[12]	
				*H. formosa* Harvey ex Kützing		N	Y	Y	N	N	[12,24]	
				*H. hawaiiana* Hernández-Kantún and A.R.Sherwood		Y	Y	Y	Y	Y	[95]	
				*H. stipitata* I.A.Abbott		N	Y	Y	Y	N	[9,12,24,95]	
			*Polyopes* J.Agardh	*P. hakalauensis* (Tilden) I.A.Abbott		Y	Y	Y	Y	N	[12,24,87,96]	
	Hildenbrandiales	Hildenbrandiaceae	*Hildenbrandia* Nardo	*H. rubra* (Sommerfelt) Meneghini		N	Y	Y	N	N	[24]	
	Nemaliales	Galaxauraceae	*Actinotrichia* Decaisne	*A. fragilis* (Forsskål) Børgesen		N	Y	Y	N	N	[9,12,24]	
			*Dichotomaria* Lamarck	*D. apiculata* (Kjellman) A.Kurihara and Masuda	*Galaxaura acuminata* Kjellman ex Butters, *G. apiculata* Kjellman	N	Y	N	N	N	[13]	
				*D. marginata* (J.Ellis and Solander) Lamarck	*Galaxaura marginata* (Ellis and Solander) J.V.Lamouroux	N	Y	Y	N	N	[9,12,24,97]	
				*D. obtusata* (J.Ellis and Solander) Lamarck	*Galaxaura obtusata* (J.Ellis and Solander) J.V.Lamouroux	N	Y	N	N	N	[9,12]	
			*Galaxaura* J.V.Lamouroux	*G. divaricata* (Linnaeus) Huisman and R.A.Townsend	*G. fasciculata* Kjellman	N	Y	Y	N	N	[12,24]	
				*G. filamentosa* R.C.Y.Chou		N	Y	Y	N	N	[12,24]	
				*G. rugosa* (J.Ellis and Solander) J.V.Lamouroux	*G. subverticillata* Kjellman	N	Y	Y	N	N	[9,12,22,97]	
			*Tricleocarpa* Huisman and Borowitzska	*T. cylindrica* (J.Ellis and Solander) Huisman and Borowitzka		N	Y	Y	N	N	[9,12,22,98]	
				*T. fastigiata* (Decaisne) Huisman, G.H.Boo and S.M.Boo	*Galaxaura fastigiata* Decaisne	N	Y	N	N	N	[13]	
				*T. fragilis* (Linnaeus) Huisman and R.A.Townsend	*T. oblongata* (Ellis and Solander) Huisman and Borowitzka	N	Y	N	N	N	[9,12,22]	
		Liagoraceae	*Akalaphycus* Huisman, I.A.Abbott and A.R.Sherwood	*A. liagoroides* (Yamada) Huisman, I.A.Abbott and A.R.Sherwood	*Stenopeltis liagoroides* (Yamada) Itono and Yoshizaki	N	Y	Y	N	N	[12,24,99]	
				*A. setchelliae* (Yamada) Huisman, I.A.Abbott and A.R.Sherwood	*Stenopeltis setchelliae* (Yamada) Itono and Yoshizaki	N	Y	Y	N	N	[12,24,99]	
			*Dermonema* Harvey ex Heydrich	*D. pulvinatum* (Grunow) Fan		N	Y	Y	N	N	[12,24]	
			*Dotyophycus* I.A.Abbott	*D. pacificus* I.A.Abbott		N	Y	N	N	N	[12]	
				*D. yamadae* (Ohmi and Itono) Abbott and Yoshizaki		N	Y	Y	N	N	[12,24]	
			*Ganonema* K.-C.Fan and Y.-C.Wang	*G. farinosum* (J.V.Lamouroux) K.-C.Fan and Y.-C.Wang		N	Y	Y	N	N	[9,12,22,100]	
				*G. papenfussii* (I.A.Abbott) Huisman, I.A.Abbott and A.R.Sherwood	*Liagora papenfussii* I.A.Abbott	N	Y	Y	Y	N	[9,12,22,24,100]	
				*G. pinnatum* (Harvey) Huisman	*Liagora pinnata* Harvey	N	Y	Y	N	N	[9,12,22,24,100]	
				*G. yoshizakii* Huisman, I.A.Abbott, and A.R.Sherwood		Y	Y	Y	Y	Y	[24,100]	
			*Gloiotrichus* Huisman and Kraft	*G. fractalis* Huisman and Kraft		N	Y	N	N	N	[101]	
			*Helminthocladia* J.Agardh	*H. rhizoidea* Doty and I.A.Abbott		Y	Y	Y	N	N	[12,24,102]	
				*H. simplex* Doty and Abbott		Y	Y	N	N	N	[12,102]	
			*Hommersandiophycus* S.-M.Lin and Huisman	*H. samaensis* (C.K.Tseng) S.-M.Lin and Huisman	*Ganonema samaense* (C.K.Tseng) Huisman, *Liagora samaensis* C.K.Tseng	N	Y	Y	N	N	[9,12,24,100]	
			*Izziella* Doty	*I. orientalis* (J.Agardh) Huisman and Schils	*Liagora orientalis* J.Agardh	N	Y	Y	N	N	[9,12,22,24]	
			*Liagora* J.V.Lamouroux	*L. albicans* J.V.Lamouroux	*L. maxima* Butters	N	Y	Y	N	N	[[9,12,22,24,99]	
				*L. boergesenii* Yamada		N	Y	Y	N	N	[12,24]	
				*L. ceranoides* J.V.Lamouroux		N	Y	Y	N	N	[9,12,22,32]	
				*L. donaldiana* I.A.Abbott and Huisman		Y	Y	Y	Y	N	[9,32,100,103]	
				*L. hawaiiana* Butters		N	Y	N	N	N	[12,22]	
				*L. julieae* Abbott and Huisman		Y	Y	Y	Y	N	[9,24,32,100,103]	
				*L. robusta* Yamada		N	Y	Y	N	N	[22,24]	
				*L. tetrasporifera* Børgesen		N	Y	N	N	N	[13]	Doubtful record.
				*L. turneri* Zanardini		N	Y	N	N	N	[22]	
			*Macrocarpus* S.-M.Lin, S.-Y.Yang and Huisman	*M. perennis* (I.A.Abbott) S.-M.Lin, S.-Y.Yang and Huisman	*Liagora perennis* I.A.Abbott	N	Y	Y	Y	N	[9,12,24,32,99]	
			*Neoizziella* Lin, S.-M., Yang, S.-Y. and Huisman	*N. divaricata* (C.K.Tseng) S.-M.Lin, S.-Y.Yang and Huisman	*Liagora divaricata* C.K.Tseng	N	Y	Y	N	N	[9,12,24,32,100]	
			*Stenopeltis* Itono and Yoshizaki	*S. gracilis* (Yamada and Tanaka) Itono and Yoshizaki		N	Y	Y	N	N	[9,12,24,32,99]	
			*Titanophycus* Huisman, G.W.Saunders and A.R.Sherwood	*T. setchellii* (Yamada) S.-M.Lin, S.-Y.Yang and Huisman	*Liagora setchellii* Yamada	N	Y	N	N	N	[12,22]	
				*T. validus* (Harvey) Huisman, G.W.Saunders and A.R.Sherwood	*Liagora valida* Harvey	N	Y	Y	N	N	[9,12,24,32,100,104]	
			*Trichogloea* Kützing	*T. lubrica* J.Agardh		N	Y	Y	N	N	[9,12,24,32,99]	
				*T. requienii* (Montagne) Kützing		N	Y	Y	N	N	[9,12,22,24]	
			*Trichogloeopsis* I.A.Abbott and Doty	*T. hawaiiana* I.A.Abbott and Doty		N	Y	N	N	N	[9,12,22]	
				*T. mucosissima* (Yamada) I.A.Abbott and Doty		N	Y	N	N	N	[12]	
		Scinaiaceae	*Scinaia* Bivona-Bernardi	*S. furcata* Zablackis		Y	Y	Y	Y	N	{9,12,24}	
				*S. hormoides* Setchell		N	Y	Y	Y	N	[9,12,24]	
				*S. huismanii* Vroom and I.A.Abbott		Y	Y	N	N	N	[9,22,105]	
		Yamadaellaceae	*Yamadaella* I.A.Abbott	*Y. caenomyce* (Decaisne) I.A.Abbott		N	Y	Y	N	N	[9,12,24,32,100]	
	Nemastomatales	Nemastomataceae	*Predaea* G.De Toni	*P. laciniosa* Kraft		N	Y	Y	N	N	[9,12,15,22,24,106]	
				*P. weldii* Kraft and I.A.Abbott		N	Y	Y	Y	N	[9,12,22,24,106]	
		Schizymeniaceae	*Platoma* Schousboe ex F.Schmitz	*P. ardreanum* Kraft and I.A.Abbott		N	Y	Y	Y	N	[9,12,24]	
			*Titanophora* (J.Agardh) Feldmann	*T. pikeana* (Dickie) Feldmann		N	Y	Y	N	N	[9,12,24]	
	Peyssonneliales	Peyssonneliaceae	*Agissea* Pestana, Lyra, Cassano and J.M.C.Nunes	*A. inamoena* (Pilger) Pestana, Lyra, Cassano and J.M.C. Nunes	*Peyssonnelia inamoena* Pilger	N	Y	Y	N	N	[12,22,24]	
				*A. orientalis* (Weber Bosse) Pestana, Lyra, Cassano and J.M.C.Nunes	*Peyssonnelia orientalis* Weber Bosse	N	Y	Y	N	N	[107]	
			*Incendia* K.R.Dixon	*I. lisianskiensis* A.R.Sherwood		Y	Y	Y	Y	Y	[107]	
			*Peyssonnelia* Decaisne	*P. conchicola* Piccone and Grunow		N	Y	Y	N	N	[12,24]	
				*P. rubra* (Greville) J.Agardh		N	Y	Y	N	N	[9,12,22,24]	
			*Ramicrusta* Zhang Derui and Zhou Jinghua	*R. hawaiiensis* A.R.Sherwood		Y	Y	Y	Y	Y	[108,109]	
				*R. lehuensis* A.R.Sherwood		Y	Y	Y	Y	Y	[108]	
			*Seiria* K.R.Dixon	*S. mesophotica* A.R.Sherwood		Y	Y	Y	Y	Y	[107]	
			*Sonderophycus* Denizot	*S. copusii* A.R.Sherwood		Y	Y	Y	Y	Y	[7]	
	Pihiellales	Pihiellaceae	*Pihiella* Huisman, Sherwood and I.A.Abbott	*P. liagoraciphila* Huisman, A.R.Sherwood and I.A.Abbott		N	Y	Y	Y	Y	[9,17]	
	Plocamiales	Plocamiaceae	*Plocamium* J.V.Lamouroux	*P. sandvicense* J.Agardh		N	Y	Y	Y	N	[9,12,22,24,32]	
	Rhodachlyales	Rhodachlyaceae	*Rhodachlya* J.A.West, J.L.Scott, K.A.West, U.Karsten, S.L.Clayden and G.W.Saunders	*R. hawaiiana* A.Kurihara, J.A.West, K.Y.Conklin and A.R.Sherwood		Y	Y	Y	Y	Y	[110]	
	Rhodophyta ordo incertae sedis	Pterocladiophilaceae	*Gelidiocolax* N.L.Gardner	*G. mammillatus* K.-C.Fan and Papenfuss		N	Y	N	N	N	[12,111]	
	Rhodymeniales	Champiaceae	*Champia* Desvaux	*C. parvula* (C.Agardh) Harvey		N	Y	Y	N	N	[9,12,22,24]	
				*C. vieillardii* Kützing		N	Y	Y	N	N	[9,12,24]	
			*Coelothrix* Børgesen	*C. irregularis* (Harvey) Børgesen		N	Y	Y	N	N	[9,12,24]	
		Faucheaceae	*Gloioderma* J.Agardh	*G. iyoense* Okamura	*Gloiocladia iyoensis* (Okamura) R.E.Norris	N	Y	Y	N	N	[12,22,24]	The taxonomic status of *Gloiocladia*, *Gloioderma*, and *Fauchea* is currently unclear and requires intensive study.
			*Leptofauchea* Kylin	*L. huawelau* E.A.Alvarado, F.P.Cabrera and A.R.Sherwood		Y	Y	Y	Y	Y	[112]	
				*L. lucida* Huisman and G.W.Saunders		N	Y	Y	Y	Y	[112]	
		Lomentariaceae	*Ceratodictyon* Zanardini	*C. intricatum* (C.Agardh) R.E.Norris	*Gelidiopsis intricata* (C.Agardh) Vickers	N	Y	N	N	N	[12,22]	
				*C. scoparium* (Montagne and Millardet) R.E.Norris	*Gelidiopsis scoparia* (Montagne and Millardet) De Toni	N	Y	Y	N	N	[9,12,24]	
				*C. variabile* (J.Agardh) R.E.Norris	*Gelidiopsis variabilis* (Greville ex J.Agardh) F.Schmitz	N	Y	N	N	N	[12,22]	
			*Yendoa* (Yendo) C.C.Santos, Lyra and J.M.C.Nunes	*Yendoa hakodatensis* (Yendo) C.C.Santos, Lyra and J.M.C.Nunes	*Lomentaria hakodatensis* Yendo	N	Y	Y	N	N	[12,22,24]	
		Rhodymeniaceae	*Botryocladia* (J.Agardh) Pfeiffer	*B. skottsbergii* (Børgesen) Levring		N	Y	Y	N	N	[9,12,22,24]	
				*B. tenuissima* W.R.Taylor		N	Y	N	N	N	[9,12,22]	
			*Chamaebotrys* Huisman	*C. boergesenii* (Weber Bosse) Huisman		N	Y	Y	N	N	[12,22,24]	
			*Chrysymenia* J.Agardh	*C. glebosa* I.A.Abbott and Littler		N	Y	N	N	N	[12,22]	
				*C. kaernbachii* Grunow		N	Y	Y	N	N	[12,24]	
				*C. okamurae* Yamada and Segawa		N	Y	Y	N	N	[12,22,24]	
				*C. procumbens* Weber Bosse		N	Y	N	N	N	[12]	
			*Coelarthrum* Børgesen	*C. cliftonii* (Harvey) Kylin		N	Y	Y	N	N	[12,22,24]	
			*Drouetia* G.De Toni	*D. coalescens* (Farlow) G.De Toni	*Halichrysis coalescens* (Farlow) R.E.Norris and A.J.K.Millar	N	Y	Y	N	N	[9,12,22,24]	
			*Erythrocolon* J.Agardh	*E. podagricum* J.Agardh		N	Y	Y	N	N	[12,24]	
			*Halichrysis* (J.Agardh) F.Schmitz	*H. irregularis* (Kützing) A.J.K.Millar		N	Y	N	N	N	[22,44]	
			*Halopeltis* J.Agardh	*H. nuahilihilia* E.A.Alvarado, F.P.Cabrera and A.R.Sherwood		Y	Y	Y	Y	Y	[112]	
			*Rhodymenia* Greville	*R. leptophylla* J.Agardh	*R. leptophylloides* E.Y.Dawson	N	Y	N	N	N	[9,12,24]	
	Sebdeniales	Sebdeniaceae	*Lesleigha* Kraft and G.W.Saunders	*L. hawaiiensis* Kraft and G.W.Saunders		N	Y	Y	Y	Y	[113]	
	Sporolithales	Sporolithaceae	*Sporolithon* Heydrich	*S. episoredion* (W.H.Adey, R.A.Townsend and Boykins) Verheij		N	Y	N	N	N	[9]	
				*S. erythraeum* (Rothpletz) Kylin		N	Y	N	N	N	[9]	
				*Sporolithon* sp.	*S. ptychoides* Heydrich	N	Y	Y	N	N	[114]	According to Richards et al. [115], Hawaiian records of *S. ptychoides* Heydrich do not represent this taxon.
	Stylonematales	Stylonemataceae	*Chroodactylon* Hansgirg	*C. ornatum* (C.Agardh) Basson	*Asterocytis ramosa* (Thwaites) Gobi ex F.Schmitz	N	Y	Y	N	N	[9,12,24]	
			*Stylonema* Reinsch	*S. alsidii* (Zanardini) K.M.Drew	*Goniotrichum alsidii* (Zanardini) M.Howe	N	Y	Y	N	N	[12,24]	
				*S. cornu-cervi* Reinsch		N	Y	N	N	N	[12,22]	
Chlorophyta	Bryopsidales	Bryopsidaceae	*Bryopsis* J.V.Lamouroux	*B. hypnoides* J.V.Lamouroux		N	Y	N	N	N	[9,14,22]	
				*B. indica* A.Gepp and E.Gepp		N	Y	N	N	N	[22]	
				*B. pennata* var. *pennata*		N	Y	N	N	N	[9,14,22]	
				*B. pennata* var. *secunda* (Harvey) Collins and Hervey		N	Y	N	N	N	[9,14]	
		Caulerpaceae	*Caulerpa* J.V.Lamouroux	*C. ambigua* Okamura	*Caulerpella ambigua* (Okamura) Prud’homme and Lokhorst	N	Y	Y	N	N	[14,22,116]	
				*C. andamanensis* (W.R.Taylor) Draisma, Prudhomme and Sauvage	*Caulerpa filicoides* var. *andamanensis* W.R.Taylor	N	Y	Y	N	N	[117]	
				*C. antoensis* Yamada		N	Y	Y	N	N	[14,22,118]	
				*C. chemnitzia* (Esper) J.V.Lamouroux	*Caulerpa peltata* J.V.Lamouroux	N	Y	Y	U	N	[22,119]	
				*C. cupressoides* (Vahl) C.Agardh		N	Y	Y	N	N	[14,22,118]	
				*C. elongata* Weber Bosse		N	Y	N	N	N	[22,119]	
				*C. elongata* f. *disticha* W.R.Taylor		N	Y	N	N	N	[14]	
				*C. filicoides* Yamada		N	Y	N	N	N	[120]	
				*C. lentillifera* J.Agardh		N	Y	N	N	N	[14]	
				*C. mexicana* Sonder ex Kützing		N	Y	Y	N	N	[118,120]	
				*C. microphysa* (Weber Bosse) Feldmann	*C. racemosa* f. *microphysa* Weber Bosse	N	Y	N	N	N	[14,22]	
				*C. nummularia* Harvey ex J.Agardh		N	Y	N	N	N	[9,14]	
				*C. racemosa* (Forsskål) J.Agardh		N	Y	Y	N	N	[9,14,22,118]	
				*C. racemosa* var. *macrophysa* (Sonder ex Kützing) W.R.Taylor	*C. macrophysa* (Sonder ex Kützing) G.Murray	N	Y	Y	N	N	[9,14,118]	
				*C. serrulata* (Forsskål) J.Agardh		N	Y	Y	N	N	[9,14,22,118]	
				*C. sertularioides* (S.G.Gmelin) M.Howe		N	Y	Y	N	N	[9,14,22,118]	
				*C. taxifolia* (M.Vahl) C.Agardh		N	Y	Y	N	N	[9,14,22,118]	
				*C. verticillata* J.Agardh		N	Y	Y	N	N	[14]	
				*C. webbiana* Montagne		N	Y	Y	N	N	[9,14,22,116,118]	
			*Caulerpa* J.V.Lamouroux	*C. webbiana* f. *disticha* Vickers		N	Y	Y	N	N	[118]	
			*Caulerpa* J.V.Lamouroux	*C. webbiana* f. *tomentella* (Harvey ex J.Agardh) Weber Bosse		N	Y	N	N	N	[119]	
		Codiaceae	*Codium* Stackhouse	*C. arabicum* Kützing		N	Y	Y	N	N	[9,14,22,116]	
				*C. campanulatum* P.C.Silva and M.E.Chacana		N	Y	N	N	N	[14]	
				*C. decorticatum* (Woodward) M.Howe		N	Y	N	N	N	[14,22]	
				*C. desultorium* P.C.Silva and M.E.Chacana		Y	Y	N	N	N	[14]	
				*C. edule* P.C.Silva		N	Y	Y	Y	N	[9,14,22,32,116]	
				*C. hawaiiense* P.C.Silva and M.E.Chacana		Y	Y	Y	N	N	[14,116]	
				*C. intermedium* P.C.Silva and M.E.Chacana		Y	Y	N	N	N	[14]	
				*C. mamillosum* Harvey		N	Y	N	N	N	[14,22]	
				*C. phasmaticum* Setchell		Y	Y	N	N	N	[14]	
				*C. picturatum* F.F.Pedroche and P.C.Silva		N	Y	N	N	N	[14]	
				*C. pomoides* J.Agardh		N	Y	N	N	N	[14]	
				*C. reediae* P.C.Silva		N	Y	N	N	N	[9,14,22]	
				*C. saccatum* Okamura		N	Y	N	N	N	[14,22]	
				*C. spongiosum* Harvey		N	Y	N	N	N	[14,22]	
				*C. subtubulosum* Okamura		N	Y	N	N	N	[14,22]	
		Derbesiaceae	*Derbesia* Solier	*D. fastigiata* W.R.Taylor		N	Y	N	N	N	[9,14,22]	
				*D. tenuissima* (Moris and De Notaris) P.Crouan and H.Crouan		N	Y	N	N	N	[9,14]	
		Dichotomosiphonaceae	*Avrainvillea* Decaisne	*A. lacerata* J.Agardh	*A. amadelpha* (Montagne) A.Gepp and E.S.Gepp	N	Y	Y	Y	Y	[9,14,121,122]	Wade [122] corrected the identification to *A. lacerata*.
		Halimedaceae	*Boodleopsis* A.Gepp and E.S.Gepp	*B. hawaiiensis* Gilbert		Y	Y	N	N	N	[14]	
			*Chlorodesmis* Harvey and Bailey	*C. caespitosa* J.Agardh	*Rhipidodesmis caespitosa* (J.Agardh) A.Gepp and E.Gepp	N	Y	Y	N	N	[9,14,22,116]	
				*C. hildebrandtii* A.Gepp and E.S.Gepp		N	Y	N	N	N	[13]	
			*Halimeda* J.V.Lamouroux	*H. copiosa* Goreau and E.A.Graham		N	Y	N	N	N	[14]	
				*H. discoidea* Decaisne		N	Y	Y	U	N	[9,14,22,123,124]	
				*H. distorta* (Yamada) Hillis-Colinvaux		N	Y	Y	N	N	[22], GenBank accession AF525647	
				*H. fragilis* W.R.Taylor		N	Y	N	N	N	[47]	
				*H. gracilis* Harvey ex J.Agardh		N	Y	Y	Y	N	[14,22,124]	
				*H. incrassata* (J.Ellis) J.V.Lamouroux		N	Y	Y	Y	N	[14,125]	
				*H. kanaloana* Vroom		N	Y	Y	Y	Y	[9,125]	
				*H. macroloba* Decaisne		N	Y	N	N	N	[14,22]	
				*H. opuntia* (Linnaeus) J.V.Lamouroux		N	Y	Y	N	N	[9,14,22]	
				*H. tuna* (J.Ellis and Solander) J.V.Lamouroux		N	Y	Y	N	N	[14]	
				*H. velasquezii* W.R.Taylor		N	Y	N	N	N	[14,22]	
			*Rhipidosiphon* Montagne	*R. javensis* Montagne		N	Y	N	N	N	[9,14,22]	
			*Siphonogramen* I.A.Abbott and Huisman	*S. abbreviatum* (W.J.Gilbert) I.A.Abbott and Huisman	*Udotea abbreviata* Gilbert	N	Y	Y	Y	N	[14,124]	
				*S. parvum* (W.J.Gilbert) I.A.Abbott and Huisman	*Pseudochlorodesmis parva* W.J.Gilbert	N	Y	N	N	N	[14,126]	
			*Udotea* J.V.Lamouroux	*U. geppiorum* Yamada		N	Y	Y	N	N	[127]	
		Ostreobiaceae	*Ostreobium* Bornet and Flahault	*O. quekettii* Bornet and Flahault		N	Y	N	N	N	[73]	
		Pseudobryopsidaceae	*Pseudobryopsis* Berthold	*P. oahuensis* Egerod		N	Y	Y	Y	N	[9,14,22,128]	
	Chaetophorales	Uronemataceae	*Uronema* Lagerheim	*U. marinum* Womersley		N	Y	N	N	N	[14,22]	
	Cladophorales	Anadyomenaceae	*Anadyomene* J.V.Lamouroux	*A. cladophoroides* (W.J.Gilbert) M.D.Guiry and A.R.Sherwood comb. nov. ^4^		Y?	Y	N	N	N	[129]	AlgaeBase lists this as a provisional record in need of confirmation.
				*A. wrightii* Harvey ex J.E.Gray		N	Y	N	N	N	[14]	
			*Microdictyon* Decaisne	*M. japonicum* Setchell		N	Y	N	N	N	[13]	
				*M. setchellianum* M.Howe		N	Y	N	N	N	[9,14,22]	
				*M. umbilicatum* (Velley) Zanardini		N	Y	Y	N	N	[9,14,22,130]	
		Boodleaceae	*Boodlea* G.Murray and De Toni	*B. composita* (Harvey) F.Brand		N	Y	Y	N	N	[9,14,22]	
				*B. montagnei* (Harvey ex J.E.Gray) Egerod		N	Y	Y	N	N	[9,14,22]	
				*B. vanbosseae* Reinbold		N	Y	N	N	N	[22]	
			*Cladophoropsis* Børgesen	*C. fasciculata* (Kjellman) Wille	*C. sundanensis* Reinbold	N	Y	N	N	N	[14]	
				*C. membranacea* (Hofman-Bang ex C.Agardh) Børgesen		N	Y	N	N	N	[14]	
			*Phyllodictyon* J.E.Gray	*P. anastomosans* (Harvey) Kraft and M.J.Wynne		N	Y	N	N	N	[9,14,22]	
		Cladophoraceae	*Chaetomorpha* Kützing	*C. aerea* (Dillwyn) Kützing		N	Y	N	N	N	[14]	
				*C. antennina* (Bory) Kützing		N	Y	N	N	N	[9,14,22]	
				*C. basiretrorsa* Setchell		N	Y	N	N	N	[9]	
				*C. brachygona* Harvey		N	Y	N	N	N	[14]	Doubtful record.
				*C. indica* (Kützing) Kützing		N	Y	N	N	N	[9,14]	
				*C. ligustica* (Kützing) Kützing	*C. capillaris* (Kützing) Børgesen	N	Y	N	N	N	[9,14]	
			*Cladophora* Kützing	*C. albida* (Nees) Kützing		N	Y	N	N	N	[120]	
				*C. fuiliginosa* Kützing	*C. catenata* Kützing	N	Y	Y	N	N	[9,130]	According to AlgaeBase “The records of *Cladophora catenata* outside the Mediterranean most likely refer to *Cladophora fuiliginosa* Kützing.” See for sequence data: https://pubmed.ncbi.nlm.nih.gov/17574874/ accessed on 3 January 2023
				*C. flexuosa* (O.F.Müller) Kützing		N	Y	N	N	N	[14,22]	
				*C. fuliginosa* Kützing	*C. luxurians* (Gilbert) Abbott and Huisman	N	Y	N	N	N	[14]	
				*C. hawaiiana* Tilden		N	Y	N	N	N	[14,22]	
				*C. laetevirens* (Dillwyn) Kützing		N	Y	N	N	N	[14,22]	
				*C. sericea* (Hudson) Kützing		N	Y	N	N	N	[9,14,22]	
				*C. socialis* Kützing	*C. patentiramea* (Montagne) Kützing	N	Y	N	N	N	[14,22]	
				*C. vagabunda* (Linnaeus) Hoek	*C. fascicularis* (Mertens ex C.Agardh) Kützing	N	Y	N	N	N	[9,14,22]	
			*Lychaete* J.Agardh	*L. sakaii* (I.A.Abbott) M.J.Wynne		N	Y	N	N	N	[120]	
				*L. dotyana* (W.J.Gilbert) M.J.Wynne	*Acrocladus dotyanus* (W.J.Gilbert) Boedeker, *Cladophora dotyana* W.J.Gilbert, *Cladophora patula* Sakai	N	Y	N	N	N	[13,14,22,47]	
				*L. herpestica* (Montagne) M.J.Wynne	*Cladophoropsis adhaerens* Gilbert, *Cladophoropsis hespestica* (Montagne) M.Howe	N	Y	N	N	N	[131,132]	
				*L. japonica* (Yamada) M.J.Wynne	*Cladophora japonica* Yamada	N	Y	N	N	N	[14,22]	
			*Pseudorhizoclonium* Boedeker	*P. africanum* (Kützing) Boedeker	*Rhizoclonium africanum* Kützing	N	Y	N	N	N	[14]	
			*Rhizoclonium* Kützing	*R. grande* Børgesen		N	Y	N	N	N	[14]	
				*R. riparium* (Roth) Harvey	*R. implexum* (Dillwyn) Kützing	N	Y	N	N	N	[14]	
		Siphonocladaceae	*Dictyosphaeria* Decaisne	*D. cavernosa* (Forsskål) Børgesen		N	Y	N	N	N	[9,14,22]	
				*D. versluysii* Weber Bosse		N	Y	N	N	N	[9,14,22]	
			*Siphonocladus* F.Schmitz	*S. tropicus* (P.Crouan and H.Crouan) J.Agardh		N	Y	Y	N	N	[9,14,22], NCBI BioProject PRJEB49977	
		Valoniaceae	*Valonia* C.Agardh	*V. aegagropila* C.Agardh		N	Y	Y	N	N	[9,14,22,130]	
				*V. trabeculata* Egerod		Y	Y	N	N	N	[9,14]	
				*V. ventricosa* J.Agardh	*Ventricaria ventricosa* (J.Agardh) J.L.Olsen and J.A.West	N	Y	N	N	N	[9,14,22]	
			*Valoniopsis* Børgesen	*V. pachynema* (G.Martens) Børgesen		N	Y	N	N	N	[14]	
	Dasycladales	Dasycladaceae	*Bornetella* Munier-Chalmas	*B. sphaerica* (Zanardini) Solms-Laubach		N	Y	N	N	N	[9,14,22]	
			*Neomeris* J.V.Lamouroux	*N. annulata* Dickie		N	Y	N	N	N	[9,14,22]	
				*N. vanbosseae* M.Howe		N	Y	N	N	N	[9,14,22]	
		Polyphysaceae	*Parvocaulis* S.Berger, U.Fettweiss, S.Gleissberg, L.B.Liddle, U.Richter, H.Sawitzky and G.C.Zuccarello	*P. clavatus* (Yamada) S.Berger, U.Fettweiss, S.Gleissberg, L.B.Liddle, U.Richter, H.Sawitzky and G.C.Zuccarello	*Acetabularia clavata* Yamada	N	Y	N	N	N	[9,14,22]	
				*P. exiguus* (Solms-Laubach) S.Berger, Fettweiss, Gleissberg, Liddle, U.Richter, Sawitzky and Zuccarello	*Acetabularia exigua* Solms-Laubach	N	Y	N	N	N	[9,14]	
				*P. parvulus* (Solms-Laubach) S.Berger, Fettweiss, Gleissberg, Liddle, U.Richter, Sawitzky and Zuccarello	*Acetabularia parvula* Solms-Laubach	N	Y	Y	N	N	[9,14,22,128]	
	Ulotrichales	Gayraliaceae	*Gayralia* K.L.Vinogradova	*G. oxysperma* (Kützing) K.L.Vinogradova ex Scagel *and al.*		N	Y	N	N	N	[9,14,22]	
		Ulotrichaceae	*Ulothrix* Kützing	*U. subflaccida* Wille		N	Y	N	N	N	[22]	
	Ulvales	Kornmanniaceae	*Neostromatella* M.J.Wynne, G.Furnari and R.Nielsen	*N. monostromatica* M.J.Wynne, G.Furnari and R.Nielsen	*Stromatella monostromatica* (P.J.L.Dangeard) Kornmann and Sahling	N	Y	N	N	N	[14]	
		Phaeophilaceae	*Phaeophila* Hauck	*P. dendroides* (P.Crouan and H.Crouan) Batters		N	Y	N	N	N	[73]	
		Ulvaceae	*Ryuguphycus* H.Kawai, T.Hanyuda and T.Kitayama	*R. kuaweuweu* (H.L.Spalding and A.R.Sherwood) H.Kawai, T.Hanyuda and T.Kitayama	*Umbraulva kuaweuweu* H.L.Spalding and A.R.Sherwood	N	Y	Y	Y	Y	[3]	
			*Ulva* Linnaeus	*U. clathrata* (Roth) C.Agardh	*Enteromorpha clathrata* (Roth) Greville	N	Y	N	N	N	[14,22,133]	According to O’Kelly et al. [133], this species is likely not found in Hawai‘i.
				*U. compressa* Linnaeus	*Enteromorpha compressa* (Linnaeus) Nees	N	Y	N	N	N	[14,22,133]	According to O’Kelly et al. [133], this species is likely not found in Hawai‘i.
				*Ulva expansa* (Setchell) Setchell and N.L.Gardner		N	Y	N	N	N	[14,22]	According to O’Kelly et al. [133], this species is likely not found in Hawai‘i.
				*U. flexuosa* Wulfen	*Enteromorpha flexuosa* (Wulfen) J.Agardh	N	Y	N	N	N	[9,14,22]	According to O’Kelly et al. [133], this species is likely not found in Hawai‘i.
				*U. iliohaha* H.L.Spalding and A.R.Sherwood		N	Y	Y	Y	Y	[3]	
				*U. intestinalis* Linnaeus	*Enteromorpha intestinalis* (Linnaeus) Nees	N	Y	N	N	N	[14,22]	According to O’Kelly et al. [133], this species is likely not found in Hawai‘i.
				*U. lactuca* Linnaeus	*U. fasciata* Delile	N	Y	Y	N	N	[9,14,22,133]	
				*Ulva linza* Linnaeus	*Enteromorpha linza* (Linnaeus) J.Agardh	N	Y	N	N	N	[14]	According to O’Kelly et al. [133], this species is likely not found in Hawai‘i.
				*U. ohiohilulu* H.L.Spalding and A.R.Sherwood		Y	Y	Y	Y	Y	[3]	
				*U. ohnoi* M.Hiraoka and S.Shimada		N	Y	Y	Y	N	[133]	
				*U. paradoxa* C.Agardh	*Enteromorpha paradoxa* (C.Agardh) Kützing, *Ulva flexuosa* subsp. *paradoxa* (C.Agardh) M.J.Wynne	N	Y	N	N	N	[14,22]	According to O’Kelly et al. [133], this species is unconfirmed in Hawai‘i.
				*U. prolifera* O.F.Müller	*Enteromorpha prolifera* (O.F.Müller) J.Agardh	N	Y	N	N	N	[14,22]	According to O’Kelly et al. [133], this species is likely not found in Hawai‘i.
				*U. reticulata* Forsskål		N	Y	N	N	N	[9,14,22]	According to O’Kelly et al. [133], this species is likely not found in Hawai‘i.
				*U. rigida* C.Agardh		N	Y	N	N	N	[14,22]	According to O’Kelly et al. [133], this species is likely not found in Hawai‘i.
				*U. taeniata* (Setchell) Setchell and N.L.Gardner		N	Y	N	N	N	[14]	According to O’Kelly et al. [133], this species is likely not found in Hawai‘i.
			*Umbraulva* E.H.Bae and I.K.Lee	*U. kaloakulau* H.L.Spalding and A.R.Sherwood		Y	Y	Y	Y	Y	[3]	
		Ulvellaceae	*Ulvella* P.Crouan and H.Crouan	*U. lens* P.Crouan and H.Crouan		N	Y	N	N	N	[14,22]	
				*U. scutata* (Reinke) R.Nielsen, C.J.O’Kelly and B.Wysor		N	Y	N	N	N	[22]	
				*U. setchellii* P.J.L.Dangeard		N	Y	N	N	N	[14]	
				*U. viridis* (Reinke) R.Nielsen, C.J.O’Kelly and B.Wysor	*Entocladia viridis* Reinke	N	Y	N	N	N	[14,22]	
	Ulvophyceae incertae sedis	Ulvophyceae familia incertae sedis	*Blastophysa* Reinke	*B. rhizopus* Reinke		N	Y	N	N	N	[9,14]	
Prasinodermatophyta	Palmophyllales	Palmophyllaceae	*Palmophyllum* Kützing	*P. crassum* (Naccari) Rabenhorst		N	Y	N	N	N	[9,14,22]	
Tracheophyta	Alismatales	Hydrocharitaceae	*Halophila* Thouars	*H. decipiens* Ostenfeld		N	Y	N	N	N	[9]	
				*H. hawaiiana* Doty and B.C.Stone		Y	Y	Y	N	N	[9,134]	
		Ruppiaceae	*Ruppia* Linnaeus	*R. maritima* Linnaeus		N	Y	Y	N	N	[9,135]	

^1^ Includes instances where a different name is or has been used to refer to the taxon in the Hawaiian Islands, and includes taxonomic synonyms as well as common and widespread misidentifications. ^2^ Yes = Y, No = No, Unknown = U. ^3^ Numbers refer to citations in the references section of this manuscript. ^4^ *Rhipidiphyllon cladophoroides* W.J.Gilbert is currently placed in a genus the type of which has been transferred to *Anadyoneme*. Thus, a new combination is necessary, as follows: *Anadyoneme cladophoroides* (W.J.Gilbert) *comb. nov.* Basionym: *Rhipidiphyllon cladophoroides* W.J.Gilbert, *Phycologia* 7: 54, figs 1, 3–8, 1969.

Due to seasonality of reproduction, or simply the infrequent nature of these events, many marine algae have heteromorphic life histories, with morphologically distinct gametophytic and sporophytic phases, and the full suite of relevant morphological characters may not be available in the specimens at hand for identification. Moreover, in recent years there have been many demonstrated instances of cryptic and pseudocryptic speciation, e.g., [136,137], as well as suspected incipient speciation—e.g., [52,138], which can provide other scenarios where reliance on morphological features can yield misidentification. Given these pitfalls of employing a strictly morphological approach to identification, greater emphasis has been placed on incorporating molecular comparisons into taxonomic identifications over the past several decades (e.g., as exemplified by recent systematic work on the brown algal genus *Lobophora*; [139,140,141]), and this is reflected in the degree of confidence in taxonomic identification that can be discerned in the current list.

The Hawaiian marine algal flora is presently comprised of 661 subgeneric taxa (652 species), which compares to the approximately 515 taxa reported for the red, green, and brown Hawaiian marine algal flora by Abbott [12] and Abbott and Huisman [14] during the most recent comprehensive compilations. Relative to other regions, the Hawaiian flora is moderately rich: it compares to the 442 species recorded from Madagascar [23], 425 from French Polynesia [142], 522 from north-western Australia [143,144], 900 from New Zealand [145], and 850 from South Africa [146]. Endemism in the Hawaiian flora is relatively low (13.1%) when compared to other organismal groups, for example: Hawaiian flowering plants (90%) and ferns (about 70% [147]). Vieira et al. [23] reported that the Malagasy seaweeds are 6.5% endemic based on their analyses to date; this is much lower compared to the reported endemism rates for Malagasy terrestrial flora and fauna (37–100%). They also noted that more comprehensive molecular surveys and cross-referencing of records are needed to clarify these figures. Indeed, the Hawaiian inventory includes several records that may be excluded or modified in the future as molecular frameworks (bolstered by intensive systematic study of smaller groups of taxa) clarify the names included here (e.g., for many of the Corallinales).

The Hawaiian archipelago is well known as a center of high endemism and biological uniqueness [2], and an urgent need exists to document the biodiversity of this unique island chain in the face of threats from habitat loss, on-going alien species introductions, invasive species and land-derived pollutants [148], changing coastlines with shoreline development, bloom-forming algae encroaching on shoreline habitat, climate change, corresponding sea-level rise and loss of coral reefs due to depth, and temperature and salinity changes [148,149]. Documentation of the baseline diversity of Hawai‘i’s marine algae and seagrasses is necessary to monitor and assess new arrivals to the State. The Hawaiian Islands are an extremely isolated island chain, clearly the most isolated on the planet, and the relatively dense human population relies heavily on imported material goods, which primarily arrive via shipping. Although research in this area has not been exhaustive, the results from a single survey of hull-fouled ships raise cause for concern. Godwin [150] surveyed eight maritime vessels on the island of O‘ahu for hull-fouling organisms and reported 14 species of red algae, nine green algae and three brown algae (a total of 26 species), of which only nine (35%) were native to the Hawaiian Islands. If these results are extrapolated to the number of vessels traveling to the Hawaiian Islands, then the potential for new algal introductions is truly staggering. At present, new records are often reported without knowledge of the vector of introduction (e.g., *Ulva ohnoi*, a “green tide” species, was reported from the coastlines of Hawai‘i for the first time in 2010 [133]), but building knowledge of the current macroalgal diversity, especially within a molecular context, will allow future introductions to be assessed more easily and accurately.

## 5. Conclusions

This compilation of 661 Hawaiian marine algae and seagrasses provides a point-in-time summary that includes a 27% increase over the last compilations from approximately 20 years ago. The fields of taxonomy and systematics have revolutionized during that interval due to the near-ubiquitous incorporation of molecular analyses, which have supported numerous new descriptions at almost all taxonomic levels, confirmation of previous morphology-based identifications, and new taxonomic combinations. With the inclusion of information about “degree of confidence” in identification derived from the use of molecular data and type specimen comparisons, we aim to bring a new level of utility to the taxonomic inventory and enable future researchers to have a solid understanding of the basis for application of each taxonomic name to the Hawaiian flora. In the face of numerous threats to biodiversity in the coming decades, it is hoped that critical inventories, such as these, will provide baseline data sets against which future changes may be compared.

## Data Availability

Not applicable.

## References

[1] Juvik S.P., Juvik J.O. (1998). Atlas of Hawaii.

[2] Zeigler A.C. (2002). Hawaiian Natural History, Ecology, and Evolution.

[3] Spalding H.L., Conklin K.Y., Smith C.M., O’Kelly C.J., Sherwood A.R. (2016). New Ulvaceae (Ulvophyceae, Chlorophyta) from mesophotic ecosystems across the Hawaiian Archipelago. J. Phycol..

[4] Cabrera F.P., Huisman J.P., Spalding H.L., Kosaki R.K., Sherwood A.R. (2022). Diversity of Kallymeniaceae (Gigartinales, Rhodophyta) associated with Hawaiian mesophotic reefs. Eur. J. Phycol..

[5] Paiano M.O., Huisman J.M., Cabrera F.P., Spalding H.L., Kosaki R.K., Sherwood A.R. (2020). *Haraldiophyllum hawaiiense* sp. nov. (Delesseriaceae, Rhodophyta): A new mesophotic genus record for the Hawaiian Islands. Algae.

[6] Sherwood A.R., Lin S.-M., Wade R.M., Spalding H.L., Smith C.M., Kosaki R.K. (2019). Characterization of *Martensia* (Delesseriaceae; Rhodophyta) from shallow and mesophotic habitats in the Hawaiian Islands: Description of four new species. Eur. J. Phycol..

[7] Sherwood A.R., Paiano M.O., Spalding H.L., Kosaki R.K. (2020). Biodiversity of Hawaiian Peyssonneliales (Rhodophyta): *Sonderophycus copusii* sp. nov., a new species from the Northwestern Hawaiian Islands. Algae.

[8] Sherwood A.R., Cabrera F.C., Kalaiwaa G.V., Fumo J.T., Spalding H.L., Kosaki K., Wagner D., Paiano M.O. (2022). A new species of *Gibsmithia* (Dumontiaceae, Rhodophyta) from mesophotic depths of the Papahānaumokuākea Marine National Monument, Hawai‘i, USA. Phycologia.

[9] Huisman J.M., Abbott I.A., Smith C.M. (2007). Hawaiian Reef Plants.

[10] Eldredge L.E., Evenhuis N.L. (2003). Hawaii’s Biodiversity: A detailed assessment of the numbers of species in the Hawaiian Islands. Bish. Mus. Occas. Pap..

[11] Sherwood A.R. (2004). Bibliographic checklist of the non-marine algae of the Hawaiian Islands. Bish. Mus. Occas. Pap. Ser..

[12] Abbott I.A. (1999). Marine Red Algae of the Hawaiian Islands.

[13] Magruder W.H., Hunt J.W. (1979). Seaweeds of Hawaii.

[14] Abbott I.A., Huisman J.M. (2004). Marine Green and Brown Algae of the Hawaiian Islands.

[15] Krayesky D.M., Norris J.N., Gabrielson P.W., Gabriel D., Fredericq S. (2009). A new order of red algae based on the Peyssonneliaceae, with an evaluation of the ordinal classification of the Florideophyceae (Rhodophyta). Proc. Biol. Soc. Wash..

[16] Le Gall L., Payri C.E., Bittner C.E., Saunders G.W. (2010). Multigene polygenetic analyses support recognition of the Sporolithales, ord. nov. Mol. Phyl. Evol..

[17] Huisman J.M., Sherwood A.R., Abbott I.A. (2003). Morphology, reproduction, and the 18S rDNA gene sequence of *Pihiella liagoraciphila* gen. et sp. nov. (Rhodophyta), the so–called ‘monosporangial discs’ associated with members of the Liagoraceae (Rhodophyta) and proposal of the Pihiellales ord. nov. J. Phycol..

[18] West J.A., Scott J.L., West K.A., Karsten U., Clayden S.L., Saunders G.W. (2008). *Rhodachlya madagascarensis* gen. et sp. nov.: A distinct acrochaetioid represents a new order and family (Rhodachlyales ord. nov., Rhodachlyaceae fam. nov.) of the Florideophyceae (Rhodophyta). Phycologia.

[19] Sherwood A.R., Huisman J.M., Paiano M.O., Williams T.M., Kosaki R.K., Smith C.M., Giuseffi L., Spalding H.L. (2020). Taxonomic determination of the cryptogenic red alga, *Chondria tumulosa* sp. nov., (Rhodomelaceae, Rhodophyta) from Papahānaumokuākea Marine National Monument, Hawai‘i, USA: A new species displaying invasive characteristics. PLoS ONE.

[20] Wade R., Spalding H., Peyton K., Foster K., Sauvage T., Ross M., Sherwood A. (2018). A new record of *Avrainvillea* cf. *erecta* (Berkeley) A. Gepp & E. S. Gepp (*Bryopsidales*, *Chlorophyta*) from urbanized estuaries in the Hawaiian Islands. Biodivers. Data J..

[21] Abbott I.A., Williamson E.H. (1974). Limu: An ethnobotanical study of some edible Hawaiian seaweeds. Bull. Pac. Trop. Bot. Gard..

[22] Tsuda R.T. (2014). Bibliographic catalogue of the marine benthic algae in the Papahānaumokuākea Marine National Monument (Northwestern Hawaiian Islands). Phytotaxa.

[23] Vieira C., N’Yeurt A.D.R., Rasoamanendrika F.A., D’Hondt S., Tran L.-A.T., Spiegel D.V.D., Kawai H., De Clerck O. (2021). Marine macroalgal biodiversity of northern Madagascar: Morpho-genetic systematics and implications of anthropic impacts for conservation. Biodivers. Cons..

[24] Sherwood A.R., Kurihara K., Conklin K.Y., Sauvage T., Presting G.G. (2010). The Hawaiian Rhodophyta Biodiversity Survey (2006-2010): A summary of principal findings. BMC Plant Biol..

[25] Sun Z., Hanyuda T., Lim P.-E., Tanaka J., Gurgel C.F.D., Kawai H. (2012). Taxonomic revision of the genus *Lobophora* (Dictyotales, Phaeophyceae) based on morphological evidence and analyses *rbcL* and *cox*3 gene sequences. Phycologia.

[26] Kraft G.T., Saunders G.W., Abbott I.A., Haroun H.J. (2004). A uniquely calcified brown alga from Hawaii: *Newhousia imbricata* gen. et sp. nov. (Dictyotales, Phaeophyceae). J. Phycol..

[27] Vieira C., Schils T., Kawai H., D’hondt S., Paiano M.O., Sherwood A.R., De Clerck O., Zubia M. (2022). Phylogenetic position of *Newhousia* (Dictyotales, Phaeophyceae) and the description of *N. sumayensis* sp. nov. from Guam. Phycologia.

[28] Hanyuda T., Arai S., Uchimura M., Prathep A., Draisma S.G.A., Phang S.M., Abbott I.A., Millar A.J.K., Kawai H., Ni-Ni-Win (2011). A taxonomic study of the genus *Padina* (Dictyotales, Phaeophyceae) including the description of four new species from Japan, Hawaii, and the Andaman Sea. J. Phycol..

[29] Beach K.S., Borgeas H.B., Smith C.M. (2006). Ecophysiological implications of the measurement of transmittance and reflectance of tropical macroalgae. Phycologia.

[30] Hanyuda T., Arai S., Uchimura M., Abbott I.A., Kawai H., Ni-Ni-Win (2008). Three new records of *Padina* in Japan based on morphological and molecular markers. Phycol. Res..

[31] Kogame K., Kurihara A., Cho G.Y., Lee K.M., Sherwood A.R., Boo S.M. (2011). *Petalonia tatewakii* sp. nov. (Scytosiphonaceae, Phaeophyceae) from the Hawaiian Islands. Phycologia.

[32] Sherwood A.R., Presting G.G. (2007). Universal primers amplify a 23S rDNA plastid marker in eukaryotic algae and cyanobacteria. J. Phycol..

[33] Lee K.M., Boo S.M., Kain (Jones) J.M., Sherwood A.R. (2013). Cryptic diversity and biogeography of the widespread brown alga *Colpomenia sinuosa* (Ectocarpales, Phaeophyceae). Bot. Mar..

[34] Santiañez W.J.E., Lee K.M., Uwai S., Kurihara A., Geraldino P.J.L., Ganzon–Fortes E.T., Boo S.M., Kogame K. (2018). Untangling nets: Elucidating the diversity and phylogeny of the clathrate brown algal genus *Hydroclathrus*, with the description of a new genus *Tronoella* (Scytosiphonaceae, Phaeophyceae). Phycologia.

[35] Mattio L., Payri C.E., Verlaque M. (2009). Taxonomic revision and geographic distribution of the subgenus *Sargassum* (Fucales, Phaeophyceae) in the western and central Pacific Islands based on morphological and molecular analyses. J. Phycol..

[36] De Wreede R.E. (1976). The phenology of three species of *Sargassum* (Sargassaceae, Phaeophyta) in Hawaii. Phycologia.

[37] Phillips N.E., Smith C.M., Morden C.W. (2005). Testing systematic concepts of *Sargassum* (Fucales, Phaeophyceae) using portions of the *rbcLS* operon. Phycol. Res..

[38] Hodgson L.M., Abbott I.A. (1992). Nearshore benthic marine algae of Cape Kina‘u, Maui. Bot. Mar..

[39] Vroom P.S., Abbott I.A. (2004). *Acrosymphyton brainardii* sp. nov. (Gigartinales, Rhodophyta) from French Frigate Shoals, northwestern Hawaiian Islands. Phycologia.

[40] Milstein D., Medeiros A.S., Oliveira E.C., Oliveira M.C. (2015). Native or introduced? A re–evaluation of *Pyropia* species (Bangiales, Rhodophyta) from Brazil based on molecular analyses. Eur. J. Phycol..

[41] Sherwood A.R. (2008). Phylogeography of *Asparagopsis taxiformis* (Bonnemaisoniales, Rhodophyta) in the Hawaiian Islands: Two mtDNA markers support three separate introductions. Phycologia.

[42] Dijoux L., Viard F., Payri C. (2014). The more we search, the more we find: Discovery of a new lineage and a new species complex in the genus *Asparagopsis*. PLoS ONE.

[43] Vroom P.S., Page K.N., Peyton K.A., Kukea-Shultz J.K. (2006). Marine algae of French Frigate Shoals, Northwestern Hawaiian Islands: Species list and biogeographic comparisons. Pac. Sci..

[44] Vroom P.S., Timmers M.A.V. (2009). Spatial and temporal comparison of algal biodiversity and benthic cover at Gardner Pinnacles, Northwestern Hawaiian Islands. J. Phycol..

[45] McDermid K.J., Abbott I.A. (2006). Deep subtidal marine plants from the Northwestern Hawaiian Islands: New perspectives on biogeography. Atoll Res. Bull..

[46] Conklin K.Y., Sherwood A.R. (2012). Molecular and morphological variation of the red alga *Spyridia filamentosa* (Ceramiales, Rhodophyta) in the Hawaiian Archipelago. Phycologia.

[47] Agegian C.R., Abbott I.A. (1985). Deep water macroalgal communities: A comparison between Penguin Bank (Hawaii) and Johnston Atoll. Proc. 5th Int. Coral Reef Congr..

[48] Won B.Y., Cho T.O., Fredericq S. (2009). Morphological and molecular characterization of species of the genus *Centroceras* (Ceramiaceae, Ceramiales), including two new species. J. Phycol..

[49] Vroom P.S. (2005). *Dasya atropurpurea* sp. Nov. (Ceramiales, Rhodophyta), a deep–water species from the Hawaiian Archipelago. Phycologia.

[50] Cassidy M.M., Schneider C.W., Saunders G.W. (2022). The *Dasya baillouviana* and *D. cryptica* complexes (Delesseriaceae, Rhodophyta) in Bermuda with three additional new species from the archipelago. J. Phycol..

[51] Wynne M.J. (2013). The Red Algal Families Delesseriaceae and Sarcomeniaceae.

[52] Sherwood A.R., Kurihara A., Conklin K.Y. (2011). Molecular diversity of Amansieae (Ceramiales, Rhodophyta) from the Hawaiian Islands: A multi-marker assessment reveals high diversity within *Amansia glomerata*. Phycol. Res..

[53] Kurihara A., Abe T., Tani M., Sherwood A.R. (2010). Molecular phylogeny and evolution of red algal parasites: A case study of *Benzaitenia*, *Janczewskia*, and *Ululania* (Ceramiales). J. Phycol..

[54] Hollenberg G.J. (1968). An account of the species of the red alga *Herposiphonia* occurring in the central and western tropical Pacific Ocean. Pac. Sci..

[55] Metti Y. (2017). *Laurencia majuscula* var. elegans (Rhodophyta, Rhodomelaceae) is reinstated to specific rank as L. elegans. Phycol. Res..

[56] Abbott I.A., Ballantine D.L., O’Doherty D.C. (2010). Morphological relationships within the genus *Lophocladia* (Rhodomelaceae, Rhodophyta) including a description of *L. kuesteri* sp. nov. from Hawai‘i. Phycologia.

[57] Hollenberg G.J. (1968). An account of the species of *Polysiphonia* of the central and western tropical Pacific Ocean. I. Oligosiphonia. Pac. Sci..

[58] Doty M.S., Gilbert W.J., Abbott I.A. (1974). Hawaiian marine algae from seaward of the algal ridge. Phycologia.

[59] Kraft G.T., Abbott I.A. (2002). The anatomy of *Neotenophycus ichthyosteus* gen. et sp. nov. (Rhodomelaceae, Ceramiales), a bizzare red algal parasite from the central Pacific. Eur. J. Phycol..

[60] Rousseau F., Gey D., Kurihara A., Maggs C.A., Martin-Lescanne J., Payri C., de Reviers B., Sherwood A.R., Le Gall L. (2017). Molecular phylogenies support taxonomic revision of three species of *Laurencia* (Rhodomelaceae, Rhodophyta), with the description of a new genus. Eur. J. Taxon..

[61] McDermid K.J., Abbott I.A. (1988). Laurencia from the Hawaiian Islands: Key, annotated list and distribution of the species. Taxonomy of Economic Seaweeds with Reference to Some Pacific and Caribbean Species.

[62] Stuercke B. (2006). An Integrated Taxonomic Assessment of North Carolina *Polysiphonia* (Ceramiales, Rhodophyta) Species. Master’s Thesis.

[63] Foslie M. (1909). Algologiske notiser VI. K. Nor. Vidensk. Selsk. Skr..

[64] Adey W.H., Townsend R.A., Boykins W.T. (1982). The crustose coralline algae (Rhodophyta: Corallinaceae) of the Hawaiian Islands. Smithson. Contrib. Mar. Sci..

[65] Kato A., Baba M., Suda S. (2011). Revision of the Mastophoroideae (Corallinales, Rhodophyta) and polyphyly in nongeniculate species widely distributed on Pacific coral reefs. J. Phycol..

[66] Woelkerling W.J., Campbell S.J. (1992). An account of the southern Australian species of *Lithophyllum* (Corallinaceae, Rhodophyta). Bull. Brit. Mus. Bot..

[67] Littler D.S., Littler M.M. (2003). South Pacific Reef Plants: A Diver’s Guide to the Plant Life of the South Pacific Coral Reefs.

[68] Foslie M. (1901). New Melobesieae. Det K. Nor. Vidensk. Selsk. Skr..

[69] Basso D., Caragnano A., Le Gall L., Rodondi G. (2015). The genus *Lithophyllum* in the north–western Indian Ocean, with description of *L. yemenense* sp. nov., *L. socotraense* sp. nov., *L. subplicatum* comb. et stat. nov., and the resumed *L. affine*, *L. kaiseri*, and *L. subreduncum* (Rhodophyta, Corallinales). Phytotaxa.

[70] Foslie M. (1903). Den botaniske samling. K. Nor. Vidensk. Selsk. Skr..

[71] Richards J.L., Kittle R.P., Abshire J.R., Fuselier D., Schmidt W.E., Gurgel C.F.D., Fredericq S. (2020). Range extension of *Mesophyllum erubescens* (Foslie) Me. Lemoine (Hapalidiales, Rhodophyta): First report from mesophotic rhodolith beds in the northwestern Gulf of Mexico offshore Louisiana and Texas, including the Flower Garden Banks National Marine Sanctuary. Check List.

[72] Womersley H.B.S. (1996). The Marine Benthic Flora of Southern Australia–Part IIIB–Gracilariales, Rhodymeniales, Corallinales and Bonnemaisoniales.

[73] Tribollet A., Langdon C., Golubic S., Atkinson M. (2006). Endolithic microflora are major primary producers in dead carbonate substrates of Hawaiian coral reefs. J. Phycol..

[74] Santelices B. (1977). A taxonomic review of Hawaiian Gelidiales (Rhodophyta). Pac. Sci..

[75] Boo G.H., Le Gall L., Rousseau F., de Reviers B., Coppejans E., Anderson R., Boo S.M. (2015). Phylogenetic relationships of *Gelidiella* (Gelidiales, Rhodophyta) from Madagascar with a description of *Gelidiella incrassata* sp. nov. Crypto. Algol..

[76] Boo G.H., Zubia M., Hughey J.R., Sherwood A.R., Fujii M.T., Boo S.M., Miller K.A. (2020). Complete mitochondrial genomes reveal population–level patterns in the widespread red alga *Gelidiella fanii* (Gelidiales, Rhodophyta). Front. Mar. Sci..

[77] Santelices B. (2004). *Parviphycus,* a new genus in the Gelidiellaceae (Gelidiales, Rhodophyta). Crypto. Algol..

[78] Kraft G.T., Saunders G.W. (2014). *Crebradomus* and *Dissimularia*, new genera in the family Chondrymeniaceae (Gigartinales, Rhodophyta) from the central, southern and western Pacific Ocean. Phycologia.

[79] Paiano M.O., Fumo J.T., Cabrera F.P., Kosaki R.K., Spalding H.L., Sherwood A.R. (2022). *Calliblepharis yasutakii* sp. nov. (Gigartinales, Rhodophyta), a new mesophotic algal species from Kapou, Papahānaumokuākea Marine National Monument, Hawai‘i, USA, and molecular evaluation of “*C. saidana*” from the Hawaiian Islands. Phytotaxa.

[80] Nauer F., Cassano V., Oliviera M.C. (2019). Description of two new Caribbean species from the *Hypnea musciformis* complex (Cystocloniaceae, Rhodophyta). Phytotaxa.

[81] Kraft G.T., Conklin K.Y., Sherwood A.R. (2014). *Tylotus laqueatus*, a new species of Dicranemataceae (Gigartinales, Rhodophyta) from the Hawaiian Islands. Phycol. Res..

[82] Abbott I.A., McDermid K.J. (2001). *Dudresnaya babbittiana* (Dumontiaceae, Gigartinales), a new red algal species from Midway Atoll, North Central Pacific. Crypto. Algol..

[83] Hoshino M., Wakeman K.C., Kitayama T., Sherwood A., Kogame K. Taxonomic study of the polyphyletic *Dudresnaya* (Dumontiaceae) with a redefinition of the family Dumontiaceae (Gigartinales, Rhodophyta). Phycologia.

[84] Gabriel D., Draisma S.G.A., Sauvage T., Schmidt W.E., Schils T., Lim P.E., Harris D.J., Fredericq S. (2016). Multilocus phylogeny reveals *Gibsmithia hawaiiensis* (Dumontiaceae, Rhodophyta) to be a species complex from the Indo-Pacific, with the proposal of *G. eilatensis* sp. nov. Phytotaxa.

[85] Gabriel D., Draisma S.G.A., Schmidt W.E., Schils T., Sauvage T., Maridakis C., Gurgel C.F.D., Harris D.J., Fredericq S. (2017). Beneath the hairy look: The hidden reproductive diversity of the *Gibsmithia hawaiiensis* complex (Dumontiaceae, Rhodophyta). J. Phycol..

[86] Sherwood A.R., Paiano M.O., Cabrera F.P., Spalding H.L., Hauck B.B., Kosaki R.K. (2021). *Ethelia hawaiiensis* (Etheliaceae, Rhodophyta), a new mesophotic marine alga from Manawai (Pearl and Hermes Atoll), Papahānaumokuākea Marine National Monument, Hawai‘i. Pac. Sci..

[87] Abbott I.A. (1998). Some new species and new combinations of marine red algae from the Central Pacific. Phycol. Res..

[88] Cabrera F.P., Huisman J.M., Spalding H.L., Kosaki R.K., Smith C.M., Sherwood A.R. (2022). Cryptic diversity in the genus *Croisettea* (Kallymeniaceae, Rhodophyta) from Hawaiian mesophotic reefs. Phycologia.

[89] Abbott I.A., McDermid K.J. (2002). On two species of *Kallymenia* (Rhodophyta: Gigartinales) from the Hawaiian Islands, Central Pacific. Pac. Sci..

[90] Leliaert F., Payo D.A., Gurgel C.F.D., Schils T., Draisma S.G.A., Saunders G.W., Kamiya M., Sherwood A.R., Lin S., Huisman J.M. (2018). Patterns and drivers of species diversity in the Indo-Pacific red seaweed *Portieria*. J. Biogeogr..

[91] Conklin K.Y., Kurihara A., Sherwood A.R. (2009). A molecular method for identification of the morphologically plastic invasive algal genera *Eucheuma* and *Kappaphycus* (Rhodophyta, Gigartinales) in Hawaii. J. Appl. Phycol..

[92] Tan J., Lim P.-E., Phang S.-M., Hong D.D., Sunarpi H., Hurtado A.Q. (2012). Assessment of Four Molecular Markers as Potential DNA Barcodes for Red Algae *Kappaphycus* Doty and *Eucheuma* J. Agardh (Solieriaceae, Rhodophyta). PLoS ONE.

[93] Conklin K.Y., O’Doherty D.C., Sherwood A.R. (2014). *Hydropuntia perplexa* n. comb. (Gracilariaceae, Rhodophyta), First Record of the Genus in Hawai‘i. Pac. Sci..

[94] Kajimura M. (1993). Morphology and taxonomic placement of *Polyopes clarionensis* Setchell et Gardner (Halymeniaceae, Rhodophyta). Bot. Mar..

[95] Hernández–Kantún J.J., Sherwood A.R., Riosmena–Rodriguez R., Huisman J.M., De Clerck O. (2012). Branched *Halymenia* species (Halymeniaceae, Rhodophyta) in the Indo–Pacific region, including descriptions of *Halymenia hawaiiana* sp. nov. and *H. tondoana* sp. nov. Eur. J. Phycol..

[96] Kawaguchi S., Shimada S., Wang H.W., Faye E., Masuda M. (2003). *Polyopes tosaensis* Kawaguchi & Masuda, sp. nov. (Halymeniaceae, Rhodophyta) from Japan. Eur. J. Phycol..

[97] Huisman J.M., Sherwood A.R., Abbott I.A. (2004). Studies of Hawaiian Galaxauraceae (Nemaliales, Rhodophyta): Large subunit rDNA gene sequences support conspecificity of *Galaxaura rugosa* and *G. subverticillata*. Crypto. Algol..

[98] Wiriyadamrikul J., Geraldino P.J.L., Huisman J.M., Lewmanomont K., Boo S.M. (2013). Molecular diversity of the calcified red algal genus *Tricleocarpa* (Galaxauraceae, Nemaliales) with the description of *T. jejuensis* and *T. natalensis*. Phycologia.

[99] Huisman J.M., Abbott I.A., Sherwood A.R. (2004). Large subunit rDNA gene sequences and reproductive morphology reveal *Stenopeltis* to be a member of the Liagoraceae (Nemaliales, Rhodophyta), with a description of *Akalaphycus* gen. nov. Eur. J. Phycol..

[100] Huisman J.M., Abbott I.A., Sherwood A.R. (2004). The Liagoraceae (Nemaliales, Rhodophyta) of the Hawaiian Islands III: The genus *Ganonema*, with a description of *G. yoshizakii* sp. nov. Phycologia.

[101] Huisman J.M., Abbott I.A. (2003). The Liagoraceae (Nemaliales, Rhodophyta) of the Hawaiian Islands I: First record of the genus *Gloiotrichus* for Hawai‘i and the Pacific Ocean. Pac. Sci..

[102] Doty M.S., Abbott I.A. (1961). Studies in the Helminthocladiaceae (Rhodophyta): *Helminthocladia*. Pac. Sci..

[103] Abbott I.A., Huisman J.M. (2003). The Liagoraceae (Nemaliales, Rhodophyta) of the Hawaiian Islands II: The species of *Liagora* with quadripartite carposporangia, including descriptions of *L. donaldiana* sp. nov. and *L. julieae* sp. nov. Phycologia.

[104] Huisman J.M., Saunders G.W., Sherwood A.R., McCarthy P.M. (2006). Recognition of *Titanophycus*, a new genus based on *Liagora valida* Harv. (Liagoraceae, Nemaliales). Algae of Australia: Nemaliales.

[105] Vroom P.S., Abbott I.A. (2004). *Scinaia huismanii* sp. nov. (Nemaliales, Rhodophyta): An addition to the exploration of the marine algae of the northwestern Hawaiian Islands. Phycologia.

[106] Gabriel D., Schils T., Neto A.I., Paramio L., Fredericq S. (2009). *Predaea feldmannii* subsp. azorica (Nemastomataceae, Nemastomatales), a new subspecies of red algae (Rhodophyta) from the Azores. Crypto. Algol..

[107] Sherwood A.R., Cabrera F.P., Spalding H.L., Alvarado E.A., Smith C.M., Hauk B.B., Matadobra S.J., Kosaki R.K., Paiano M.O. (2021). Biodiversity of Hawaiian Peyssonneliales (Peyssonneliaceae, Rhodophyta): New species in the genera *Incendia* and *Seiria*. Phytotaxa.

[108] Sherwood A.R., Paiano M.O., Wade R.M., Cabrera F.C., Spalding H.L., Kosaki R.K. (2021). Biodiversity of Hawaiian Peyssonneliales (Rhodophyta). 1. Two new species in the genus *Ramicrusta* from Lehua Island. Pac. Sci..

[109] Grady B.W., Kittle R.P., Pugh A., Lamson M.R., Richards J.L., Fredericq S., McDermid K.J., Allen Q., Asner G.P. (2022). Long-term ecological monitoring of reefs on Hawai’i Island (2003-2020): Characterization of a common cryptic crust, *Ramicrusta hawaiiensis* (Peyssonneliales, Rhodophyta). Front. Mar. Sci..

[110] Kurihara A., West J.A., Conklin K.Y., Sherwood A.R. (2012). A second species of *Rhodachlya* (Rhodachlyales, Rhodophyta) from Hawaii, with a description of *R. hawaiiana* sp. nov. Crypto. Algol..

[111] Fan K.-C., Papenfuss G.F. (1959). Red algal parasites occurring on members of the Gelidiales. Madroño.

[112] Alvarado E.A., Cabrera F.P., Paiano M.O., Fumo J.T., Spalding H.L., Smith C.M., Leonard J.C., Lopes Jr. K., Kosaki R.K., Sherwood A.R. (2022). Unveiling mesophotic diversity in Hawai‘i: Two new species in the genera *Halopeltis* and *Leptofauchea* (Rhodymeniales, Rhodophyta). ALGAE.

[113] Kraft G.T., Saunders G.W. (2011). Taxonomic and molecular studies of the family Sebdeniaceae (Sebdeniales, Rhodophyta): New species of *Lesleigha* gen. nov. and *Crassitegula* from Hawaii, east Asia and Lord Howe Island. Eur. J. Phycol..

[114] Verheij E. (1993). The genus *Sporolithon* (Sporolithaceae fam. nov., Corallinales, Rhodophyta) from the Spermonde Archipelago, Indonesia. Phycologia.

[115] Richards J.L., Sauvage T., Schmidt W.E., Fredericq S., Hughey J.R., Gabrielson P.W. (2017). The coralline genera *Sporolithon* and *Heydrichia* (Sporolithales, Rhodophyta) clarified by sequencing type material of their generitypes and other species. J. Phycol..

[116] Wade R.M., Sherwood A.R. (2017). Molecular determination of kleptoplast origins from the sea slug *Plakobranchus ocellatus* (Sacoglossa, Gastropoda) reveals cryptic bryopsidalean (Chlorophyta) diversity in the Hawaiian Islands. J. Phycol..

[117] Draisma S.G.A., van Reine W.F.P., Sauvage T., Belton G.S., Gurgel C.F.D., Lim P.E., Phang S.M. (2014). A re–assessment of the infra–generic classification of the genus *Caulerpa* (Caulerpaceae, Chlorophyta) inferred from a time–calibrated molecular phylogeny. J. Phycol..

[118] Sauvage T. (2010). Molecular Revision of the Core *Caulerpa* in the Hawaiian Archipelago. Master’s Thesis.

[119] Hodgson L.M., Pham Huu T., Lewmanomont K., McDermid K.J., Abbott I.A., McDermid K.J. (2004). Annotated checklist of species of *Caulerpa* and *Caulerpella* (Bryopsidales, Caulerpaceae) from Vietnam, Thailand and the Hawaiian Islands. Taxonomy of Economic Seaweeds with Reference to the Pacific and other Locations, Volume 9.

[120] Spalding H.L. (2012). Ecology of Mesophotic Macroalgae and *Halimeda kanaloana* Meadows in the Main Hawaiian Islands. Ph.D. Dissertation.

[121] Brostoff W.N. (1989). *Avrainvillea amadelpha* (Codiales, Chlorophyta) from Oahu, Hawaii. Pac. Sci..

[122] Wade R.M. (2019). An Algivorous Sea Slug as a Novel Sampling Tool and Its Implications for Algal Diversity, Herbivore Ecology, and Invasive Species Tracking. Ph.D. Dissertation.

[123] Verbruggen H., Clerck O.D., Schils T., Kooistra W.H., Coppejans E. (2005). Evolution and phylogeography of *Halimeda* section *Halimeda* (Bryopsidales, Chlorophyta). Mol. Phylo. Evol..

[124] Verbruggen H., Tyberghein L., Pauly K., Vlaeminck C., Van Nieuwenhuyze K., Kooistra W.H.C.F., Leliaert F., De Clerck O. (2009). Macroecology meets macroevolution: Evolutionary niche dynamics in the seaweed *Halimeda*. Glob. Ecol. Biogeogr..

[125] Verbruggen H., De Clerck O., N’Yeurt A.D.R., Spalding H., Vroom P. (2006). Phylogeny and taxonomy of *Halimeda incrassata*, including descriptions of *H. kanaloana* and *H. heteromorpha* spp. nov. (Bryopsidales, Chlorophyta). Eur. J. Phycol..

[126] Tsuda R.T., Spalding H.L., Sherwood A.R. (2015). New species records of marine benthic algae in the Papahānaumokuākea Marine National Monument (Northwestern Hawaiian Islands). Bish. Mus. Occ. Pap..

[127] Sauvage T., Ballantine D.L., Peyton K.A., Wade R.M., Sherwood A.R., Keeley S., Smith C. (2019). Molecular confirmation and morphological reassessment of *Udotea geppiorum* (Bryopsidales, Chlorophyta) with ecological observations of mesophotic meadows in the Main Hawaiian Islands. Eur. J. Phycol..

[128] Sauvage T., Schmidt W.E., Suda S., Fredericq S. (2016). A metabarcoding framework for facilitated survey of endolithic phototrophs with *tuf*A. BMC Ecol..

[129] Gilbert W.J. (1968). *Rhipidiphyllon cladophoroides*, a New Marine Green Alga from Hawaii. Phycologia.

[130] Leliaert F., De Clerck O., Verbruggen H., Boedeker C., Coppejans E. (2007). Molecular phylogeny of the Siphonocladales. Mol. Phyl. Evol..

[131] Gilbert W.J. (1962). Contributions to the marine Chlorophyta of Hawaii. I. Pac. Sci..

[132] Leliaert F., Coppejans E. (2006). A revision of *Cladophoropsis* Børgesen (Siphonocladales, Chlorophyta). Phycologia.

[133] O’Kelly C.J., Kurihara A., Shipley T.C., Sherwood A.R. (2010). Molecular assessment of *Ulva* spp. (Ulvophyceae, Chlorophyta) in the Hawaiian Islands. J. Phycol..

[134] Waycott M., Freshwater D.W., York R.A., Calladine A., Kenworthy W.J. (2002). Evolutionary trends in the seagrass genus *Halophila* (Thouars): Insights from molecular phylogeny. Bull. Mar. Sci..

[135] Colwell B.A., Kittle R.P., Corpuz R.L., McDermid K.J. (2021). Molecular systematics of the native seagrass, *Ruppia* cf. *maritima* (Ruppiaceae, Alismatales), on Hawai‘i Island. Pac. Sci..

[136] Neiva J., Serrão E.A., Anderson L., Raimondi P.T., Martins N., Gouveia L., Paulino C., Coelho N.C., Miller K.A., Reed D.C. (2017). Cryptic diversity, geographical endemism and allopolyploidy in NE Pacific seaweeds. BMC Evol. Biol..

[137] Nalley E.M., Donahue M.J., Toonen R.J. (2022). Metabarcoding as a tool to examine cryptic algae in the diets of two common grazing surgeonfishes, *Acanthurus triostegus* and *A. nigrofuscus*. Envir. DNA.

[138] Roberson L.M., Coyer J.A. (2004). Variation in blade morphology of the kelp *Eisenia arborea*: Incipient speciation due to local water motion?. Mar. Ecol. Prog. Ser..

[139] Vieira C., Rasoamanendrika F.A., Zubia M., Bolton J.J., Anderson R.J., Engelen A.H., D’Hondt S., Leliaert F., Payri C., Kawai H. (2021). *Lobophora* (Dictyotales, Phaeophyceae) from the Western Indian Ocean: Diversity and biogeography. S. Afr. J. Bot..

[140] Vieira C., Steen F., D’hondt S., Bafort Q., Tyberhein L., Fernandez-García C., Wysor B., Tronholm A., Mattio L., Payri C. (2021). Global biogeography and diversification of a group of brown seaweeds (Phaeophyceae) driven by clade-specific evolutionary processes. J. Biogeog..

[141] Vieira C., De Clerck O., N’Yeurt A.R., D’Hondt S., Millet L., Kim M.S., Payri C., Zubia M. (2022). Diversity, systematics and biogeography of French Polynesian *Lobophora* (Dictyotales, Phaeophyceae). Eur. J. Phycol..

[142] Payri C.E., N’Yeurt A.D.R. (1997). A Revised Checklist of Polynesian Benthic Marine Algae. Aust. Syst. Bot..

[143] Huisman J.M. (2015). Algae of Australia: Marine Benthic Algae of North-western Australia, 1. Green and Brown Algae.

[144] Huisman J.M. (2018). Algae of Australia: Marine Benthic Algae of North-western Australia, 2. Red Algae.

[145] Nelson W. (2013). New Zealand Seaweeds: An Illustrated Guide.

[146] De Clerck O., Bolton J.J., Anderson R.J., Coppejans E. (2005). Guide to the Seaweeds of Kwazulu-Natal.

[147] Keeley S.C., Funk V.A., Bramwell D., Caujapé-Castells J. (2011). Origin and evolution of Hawaiian endemics: New patterns revealed by molecular phylogenetic studies. The Biology of Island Floras.

[148] Smith J.E., Hunter C.L., Smith C.M. (2002). Distribution and reproductive characteristics of nonindigenous and invasive marine algae in the Hawaiian islands. Pac. Sci..

[149] Webster J.M., Clague D.A., Riker-Coleman K., Gallup C., Braga J.C., Potts D., Moore J.G., Winterer E.L., Paull C.K. (2004). Drowning of the −150 m reef off Hawaii: A casualty of global meltwater pulse 1A?. Geology.

[150] Godwin L.S. (2003). Hull fouling of maritime vessels as a pathway for marine species invasions to the Hawaiian Islands. Biofouling.

